# Supplementary figures and images for: Activating SRC/MAPK signaling via 5-HT1A receptor contributes to the effect of vilazodone on improving thrombocytopenia
Source: eLife. 2024 Apr 4;13:RP94765. doi: 10.7554/eLife.94765 (PMC10994662; doi:10.7554/eLife.94765)

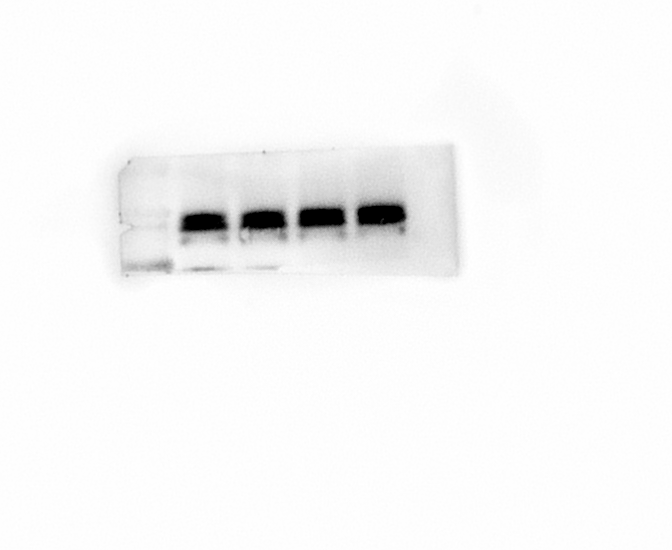

Supplement: Figure 7—source data 1. [file elife-94765-fig7-data1.zip › Figure 7-soure data 1/RAS/RAS-2.tif]

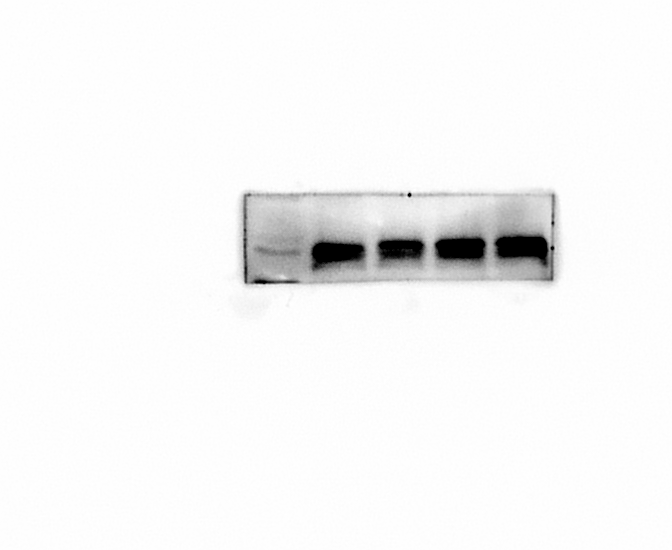

Supplement: Figure 7—source data 1. [file elife-94765-fig7-data1.zip › Figure 7-soure data 1/RAS/RAS_3.tif]

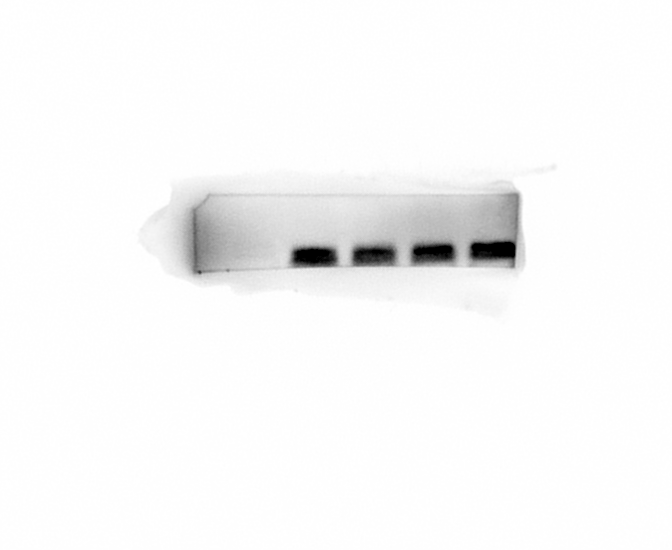

Supplement: Figure 7—source data 1. [file elife-94765-fig7-data1.zip › Figure 7-soure data 1/RAS/RAS_1.tif]

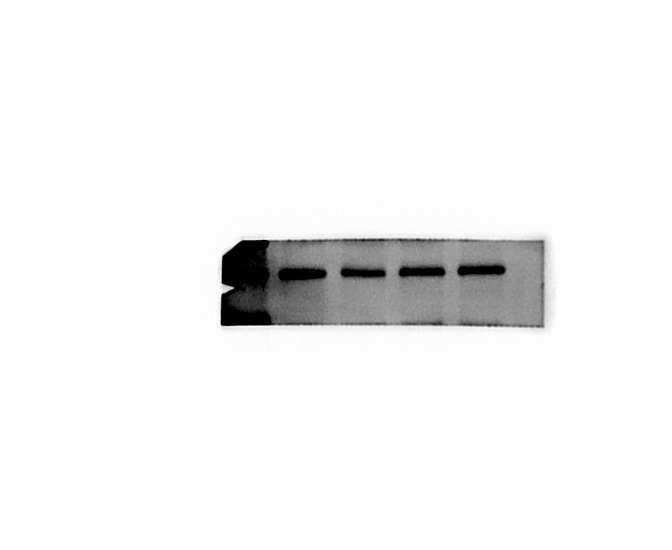

Supplement: Figure 7—source data 1. [file elife-94765-fig7-data1.zip › Figure 7-soure data 1/RAS/GAPDH_3.tif]

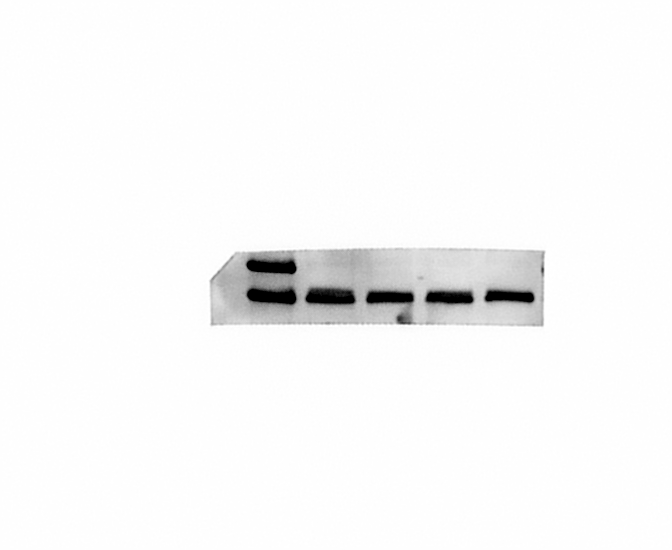

Supplement: Figure 7—source data 1. [file elife-94765-fig7-data1.zip › Figure 7-soure data 1/RAS/GAPDH_1.tif]

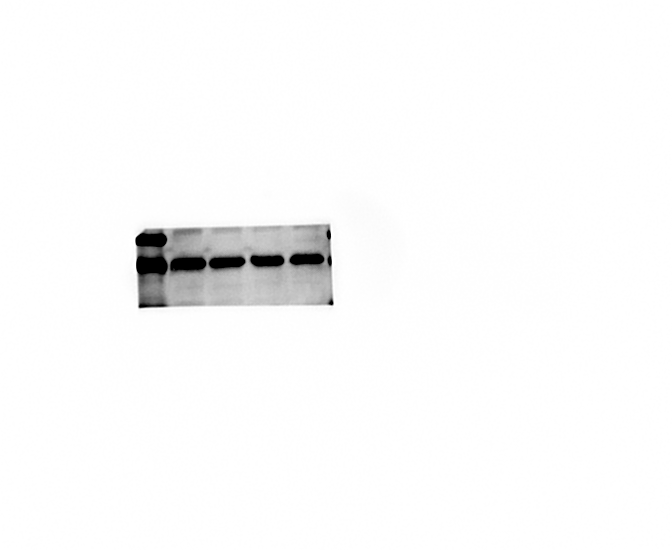

Supplement: Figure 7—source data 1. [file elife-94765-fig7-data1.zip › Figure 7-soure data 1/RAS/GAPDH-2.tif]

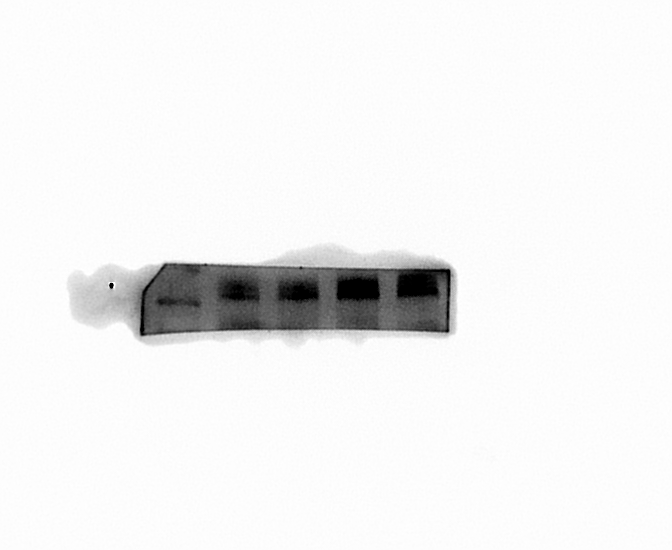

Supplement: Figure 7—source data 1. [file elife-94765-fig7-data1.zip › Figure 7-soure data 1/NFE2/NFE2_4.tif]

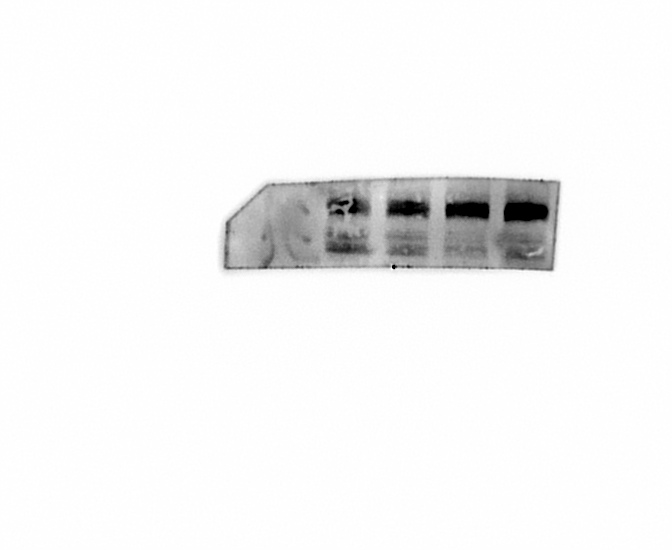

Supplement: Figure 7—source data 1. [file elife-94765-fig7-data1.zip › Figure 7-soure data 1/NFE2/NFE2_1.tif]

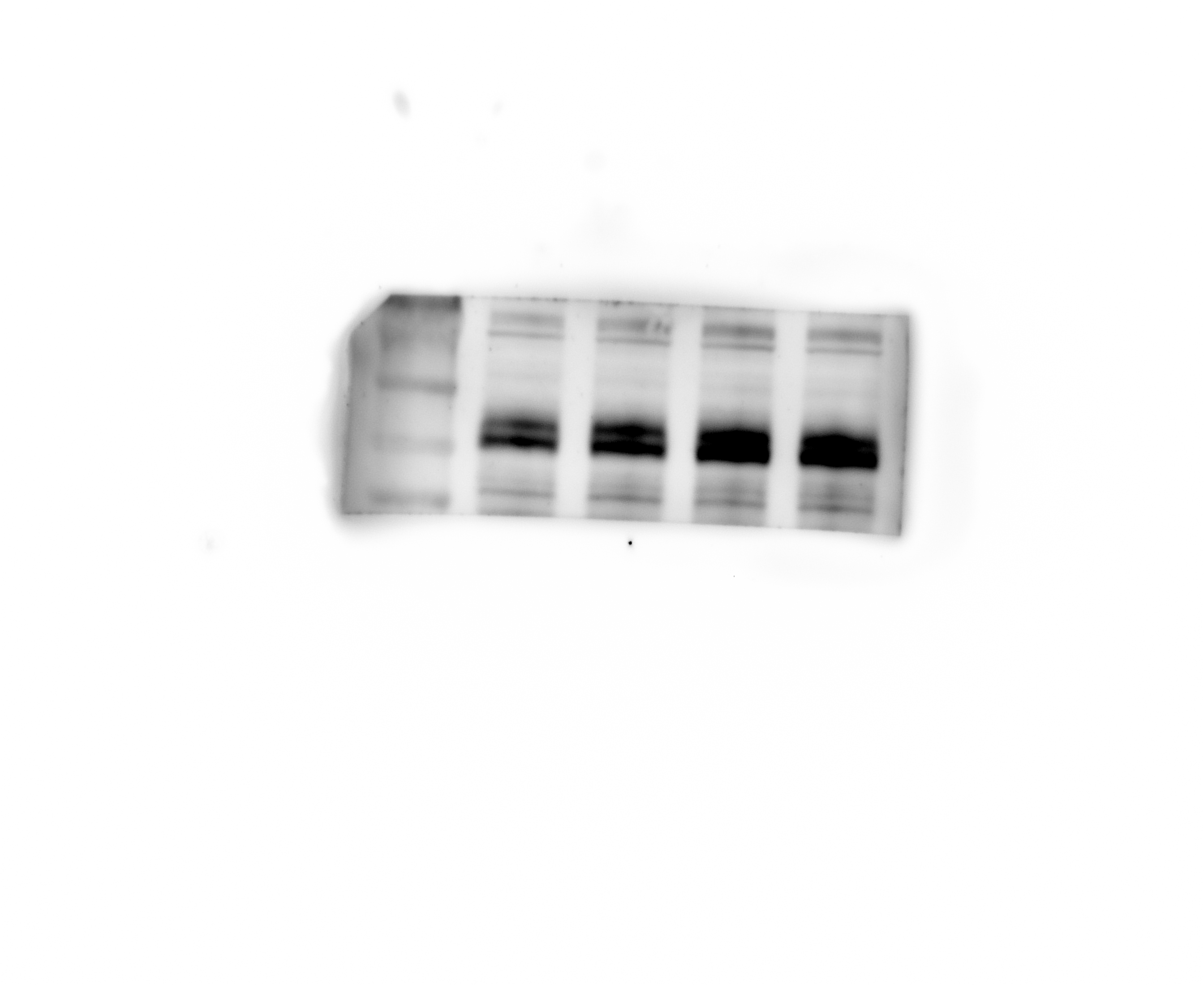

Supplement: Figure 7—source data 1. [file elife-94765-fig7-data1.zip › Figure 7-soure data 1/NFE2/NFE2-3.tif]

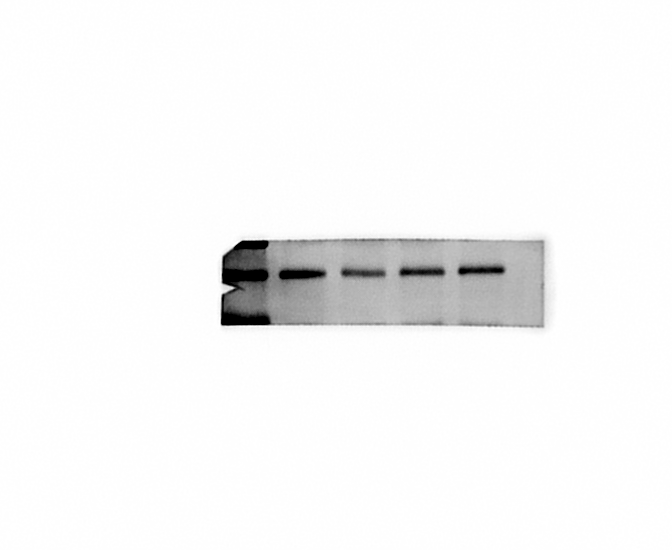

Supplement: Figure 7—source data 1. [file elife-94765-fig7-data1.zip › Figure 7-soure data 1/NFE2/GAPDH_1.tif]

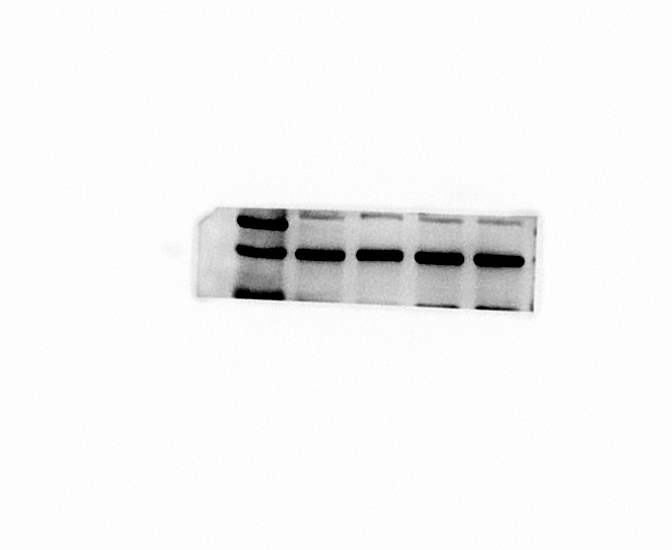

Supplement: Figure 7—source data 1. [file elife-94765-fig7-data1.zip › Figure 7-soure data 1/NFE2/GAPDH_4.tif]

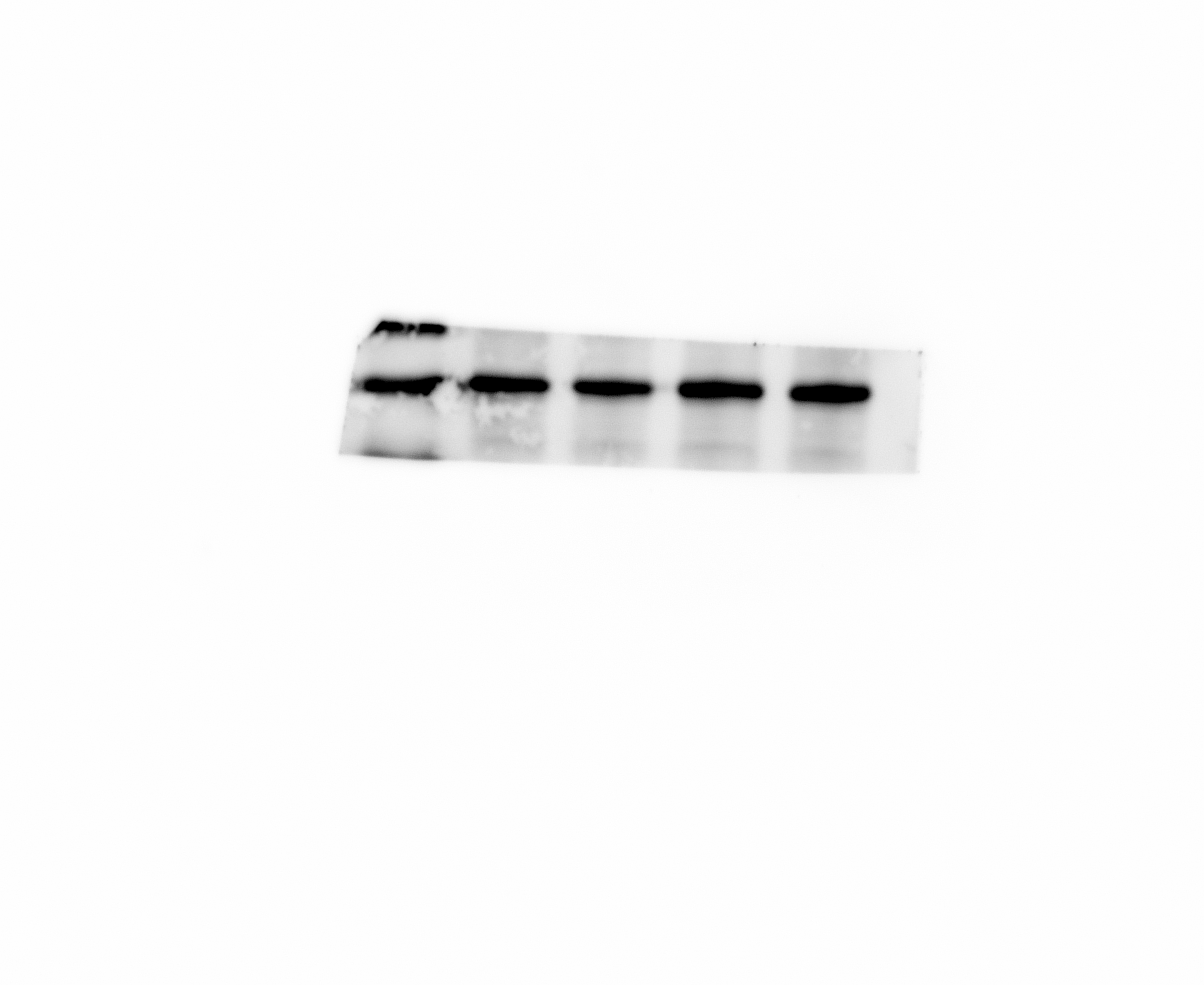

Supplement: Figure 7—source data 1. [file elife-94765-fig7-data1.zip › Figure 7-soure data 1/NFE2/GAPDH-3.tif]

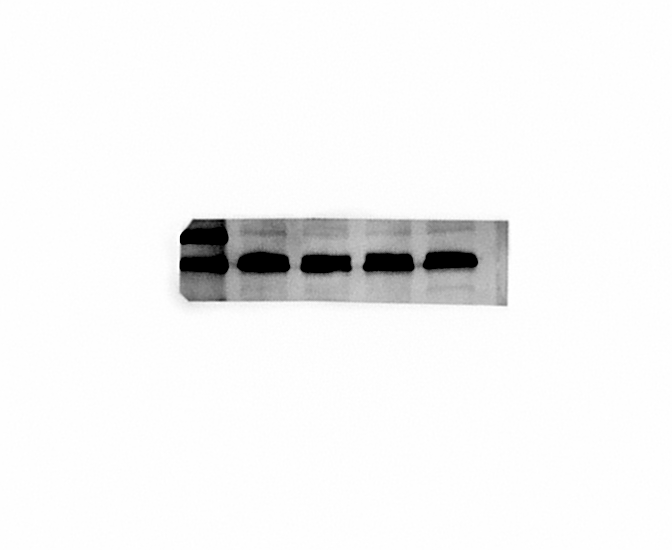

Supplement: Figure 7—source data 1. [file elife-94765-fig7-data1.zip › Figure 7-soure data 1/NFE2/GAPDH_4_230228_170318_00.01.000_1_16111.tif]

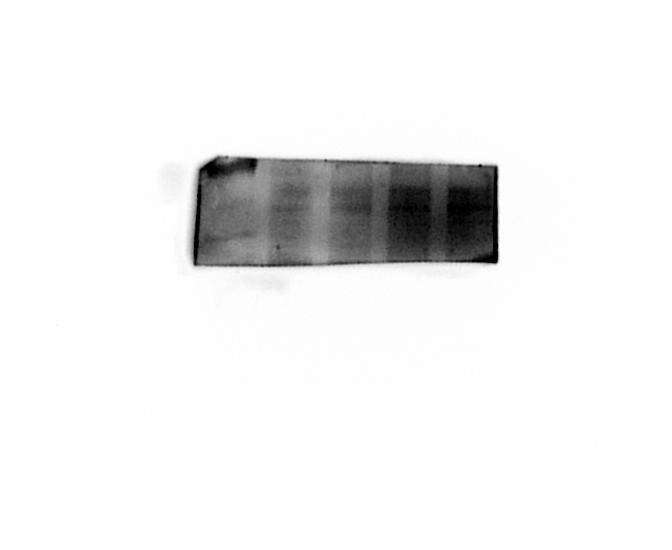

Supplement: Figure 7—source data 1. [file elife-94765-fig7-data1.zip › Figure 7-soure data 1/RUNX1/RUNX1_1.tif]

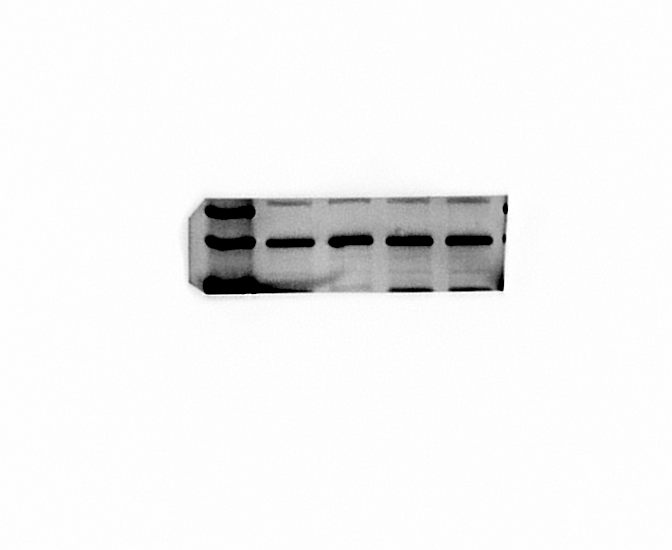

Supplement: Figure 7—source data 1. [file elife-94765-fig7-data1.zip › Figure 7-soure data 1/RUNX1/GAPDH_2.tif]

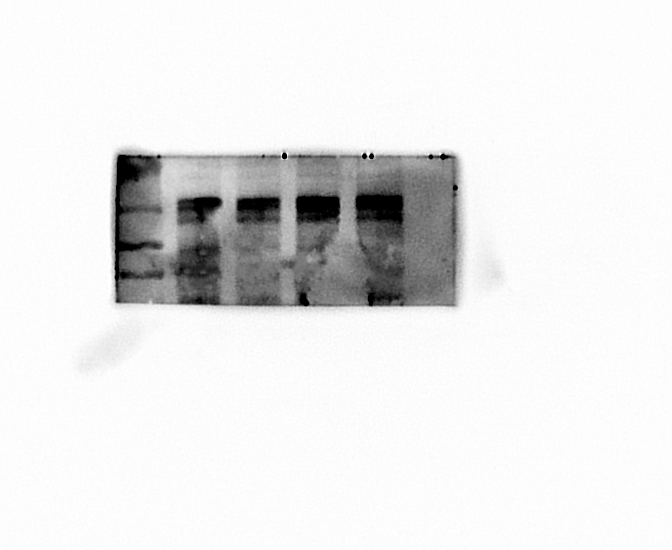

Supplement: Figure 7—source data 1. [file elife-94765-fig7-data1.zip › Figure 7-soure data 1/RUNX1/RUNX1_3.tif]

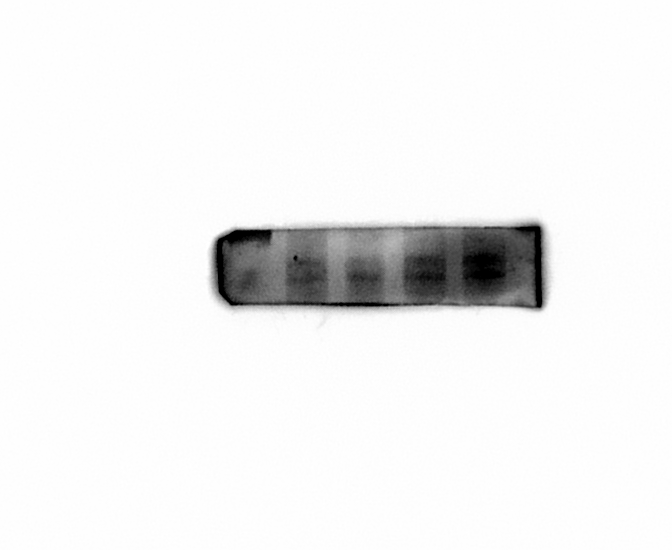

Supplement: Figure 7—source data 1. [file elife-94765-fig7-data1.zip › Figure 7-soure data 1/RUNX1/RUNX1_2.tif]

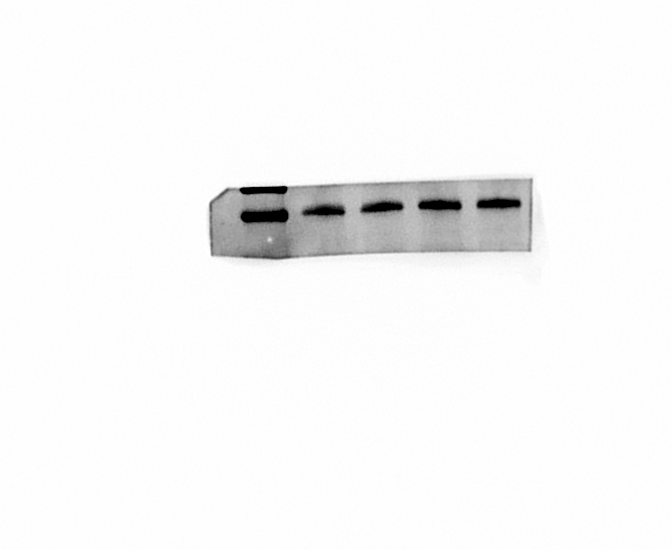

Supplement: Figure 7—source data 1. [file elife-94765-fig7-data1.zip › Figure 7-soure data 1/RUNX1/GAPDH_1.tif]

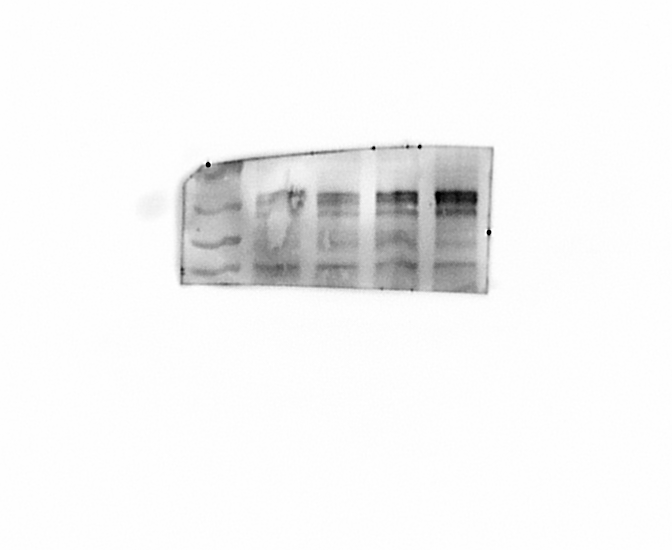

Supplement: Figure 7—source data 1. [file elife-94765-fig7-data1.zip › Figure 7-soure data 1/RUNX1/RUNX1-4.tif]

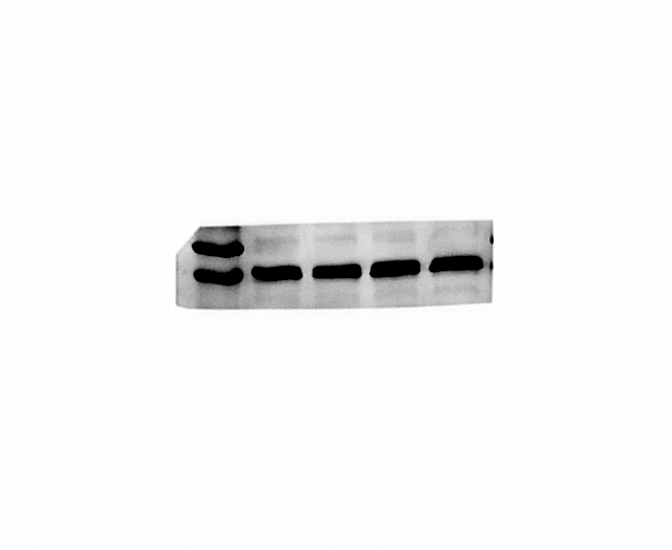

Supplement: Figure 7—source data 1. [file elife-94765-fig7-data1.zip › Figure 7-soure data 1/RUNX1/GAPDH-4.tif]

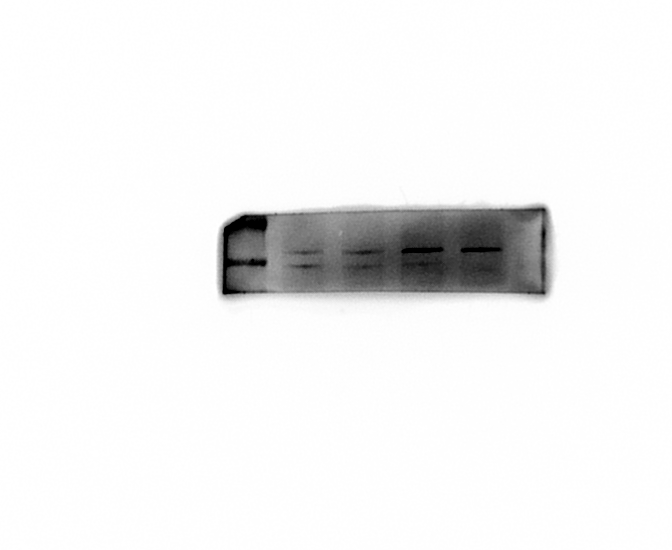

Supplement: Figure 7—source data 1. [file elife-94765-fig7-data1.zip › Figure 7-soure data 1/FOS/FOS-1.tif]

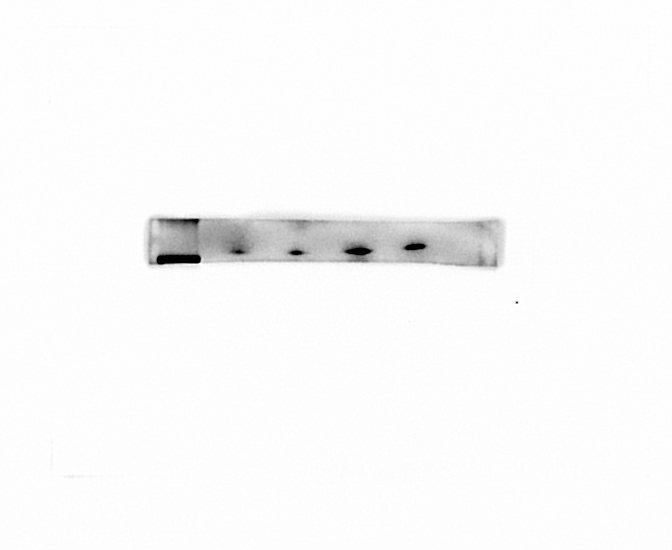

Supplement: Figure 7—source data 1. [file elife-94765-fig7-data1.zip › Figure 7-soure data 1/FOS/FOS-3.tif]

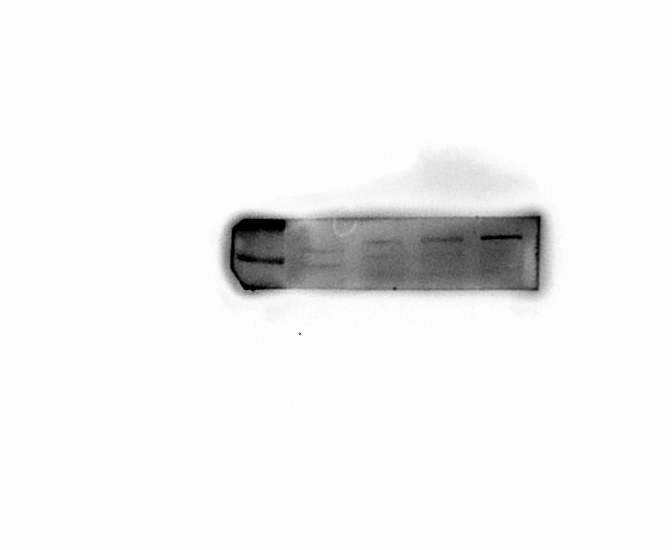

Supplement: Figure 7—source data 1. [file elife-94765-fig7-data1.zip › Figure 7-soure data 1/FOS/FOS-2.tif]

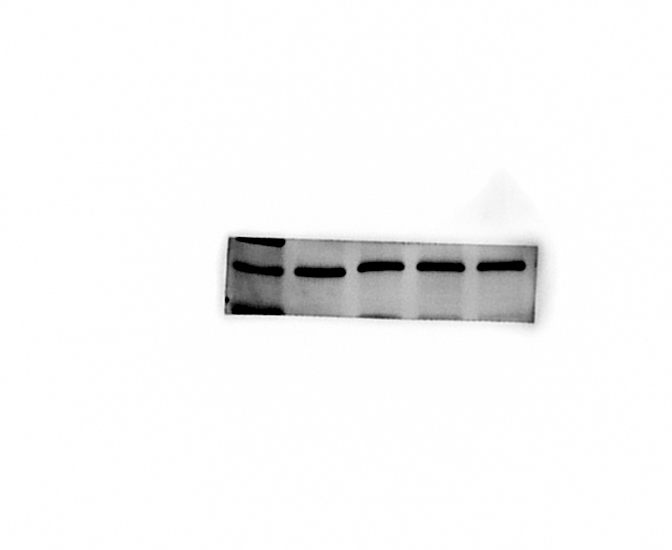

Supplement: Figure 7—source data 1. [file elife-94765-fig7-data1.zip › Figure 7-soure data 1/FOS/GAPDH-2.tif]

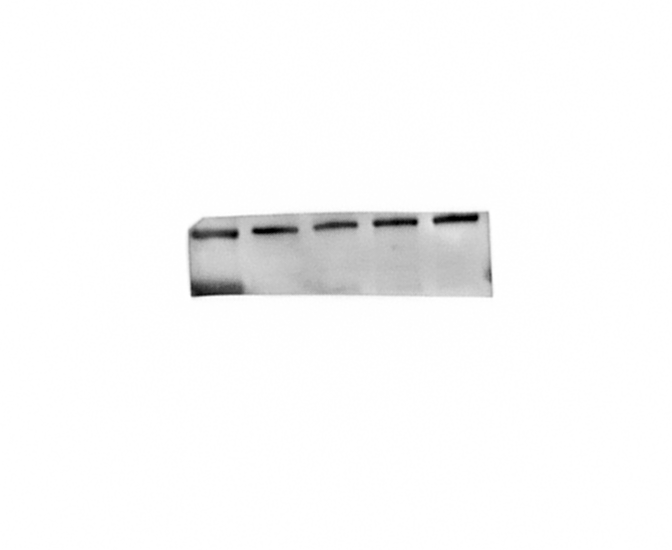

Supplement: Figure 7—source data 1. [file elife-94765-fig7-data1.zip › Figure 7-soure data 1/FOS/GAPDH-3.tif]

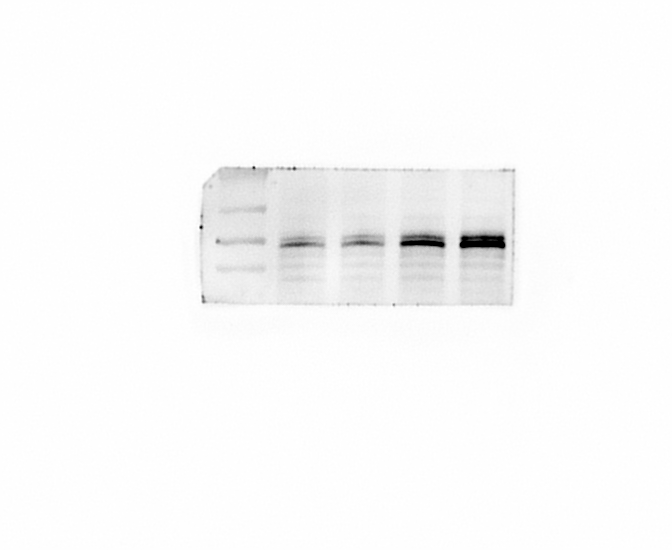

Supplement: Figure 7—source data 1. [file elife-94765-fig7-data1.zip › Figure 7-soure data 1/ERK/P-ERK-1.tif]

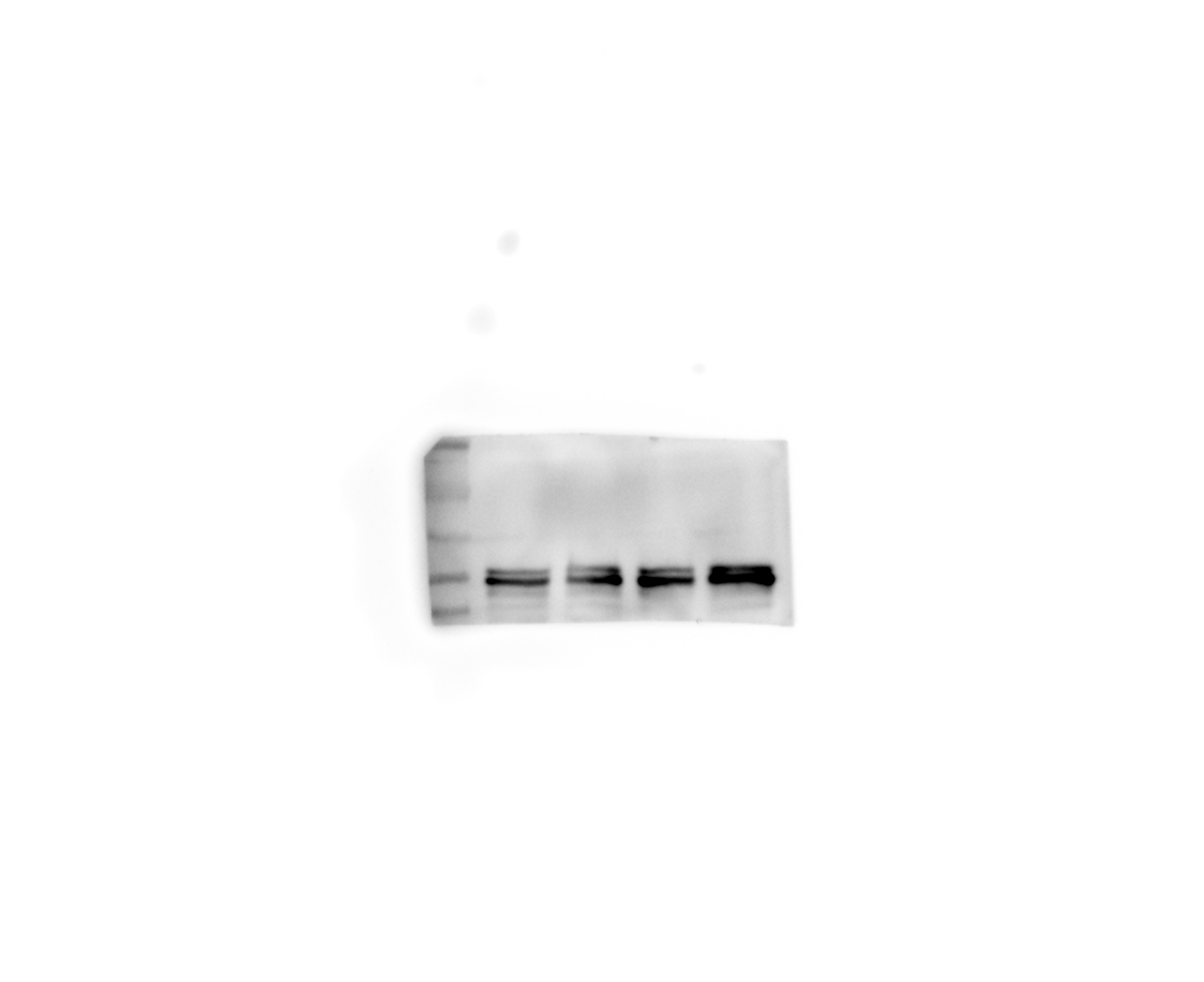

Supplement: Figure 7—source data 1. [file elife-94765-fig7-data1.zip › Figure 7-soure data 1/ERK/P-ERK-2.Tif]

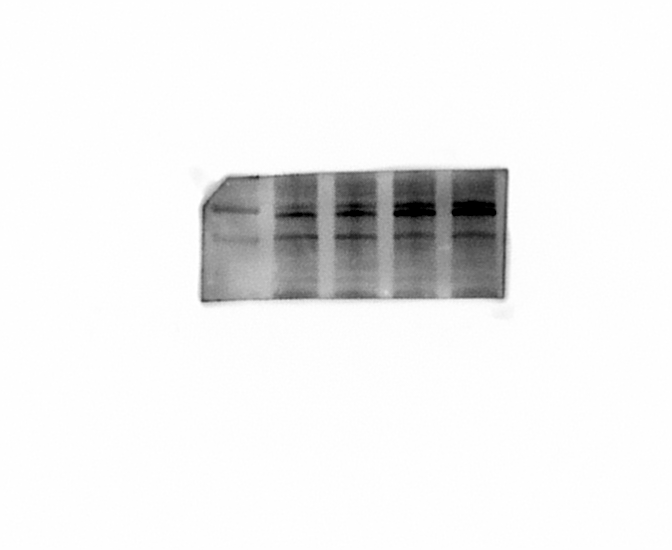

Supplement: Figure 7—source data 1. [file elife-94765-fig7-data1.zip › Figure 7-soure data 1/ERK/P-ERK-3.tif]

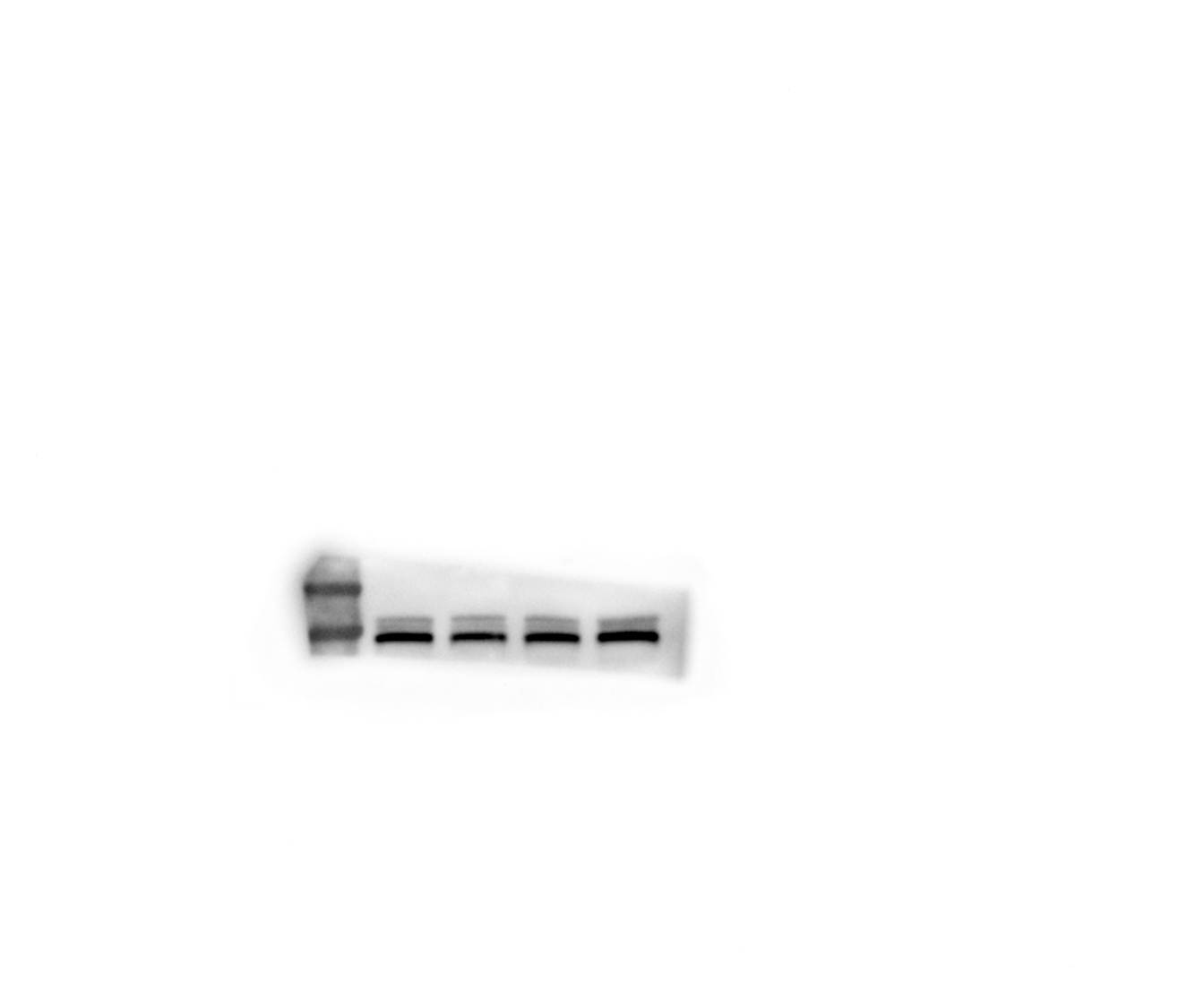

Supplement: Figure 7—source data 1. [file elife-94765-fig7-data1.zip › Figure 7-soure data 1/ERK/ERK-1.Tif]

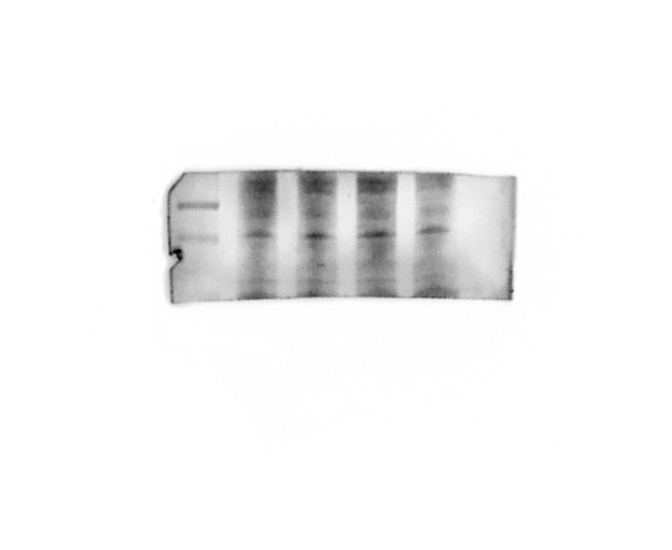

Supplement: Figure 7—source data 1. [file elife-94765-fig7-data1.zip › Figure 7-soure data 1/ERK/ERK-3.tif]

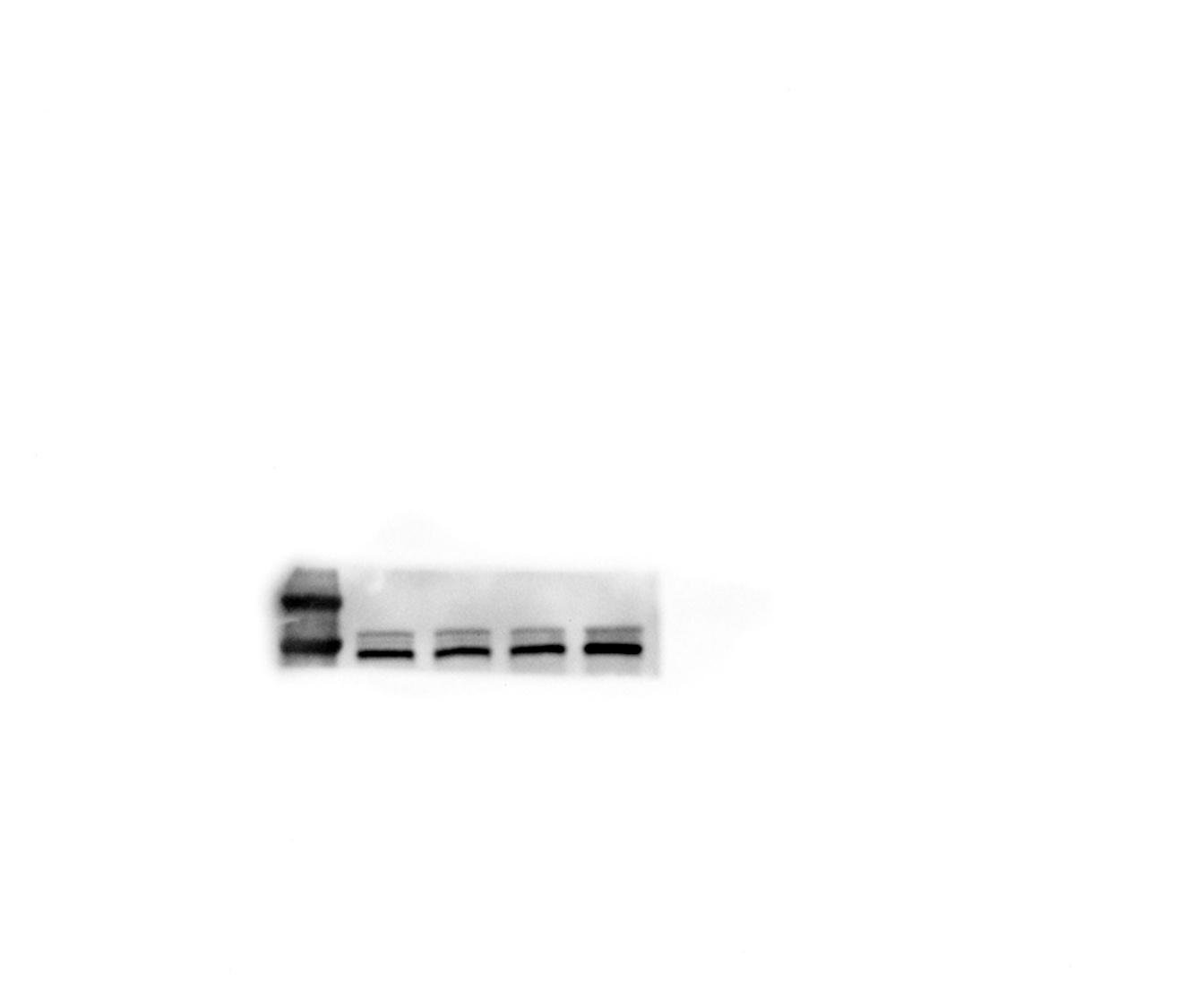

Supplement: Figure 7—source data 1. [file elife-94765-fig7-data1.zip › Figure 7-soure data 1/ERK/ERK-2.Tif]

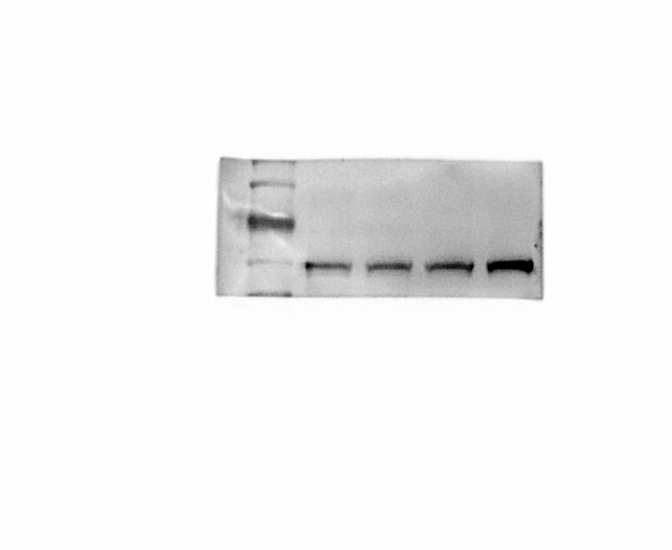

Supplement: Figure 7—source data 1. [file elife-94765-fig7-data1.zip › Figure 7-soure data 1/EGR1/EGR1_1_230323_113041_00.03.000_1_16040.tif]

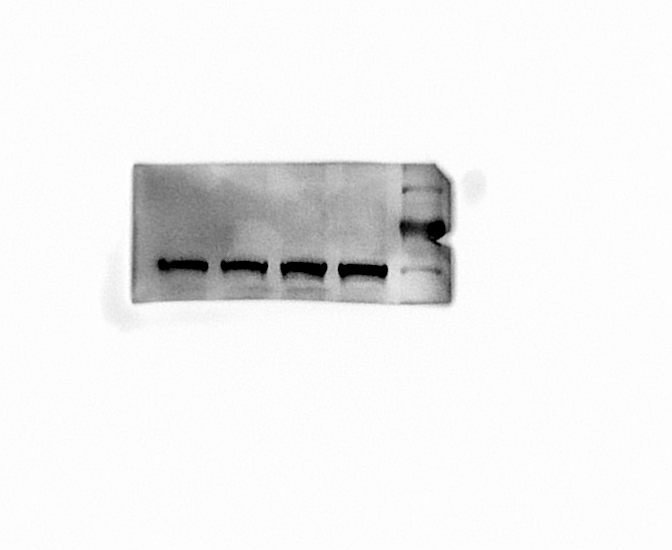

Supplement: Figure 7—source data 1. [file elife-94765-fig7-data1.zip › Figure 7-soure data 1/EGR1/EGR1_3_230323_113459_00.03.000_1_9106.tif]

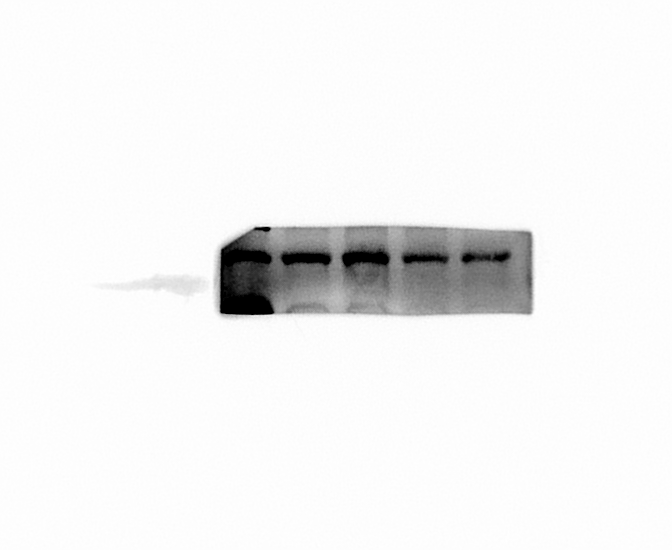

Supplement: Figure 7—source data 1. [file elife-94765-fig7-data1.zip › Figure 7-soure data 1/EGR1/GAPDH_3.tif]

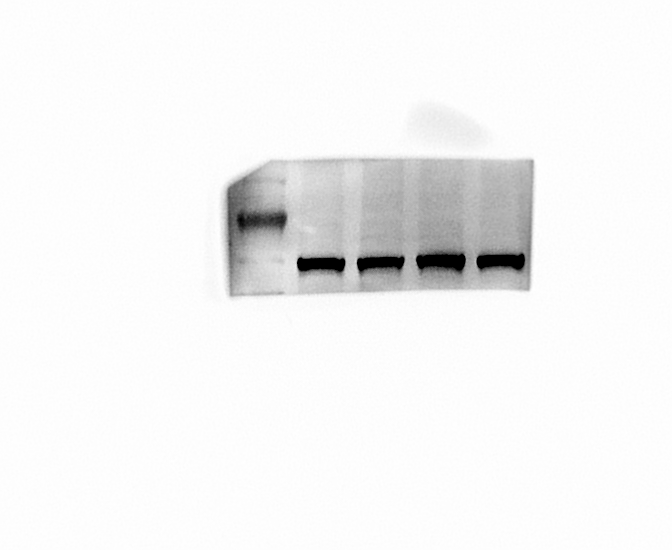

Supplement: Figure 7—source data 1. [file elife-94765-fig7-data1.zip › Figure 7-soure data 1/EGR1/EGR1_2_230323_113228_00.02.000_1_12239.tif]

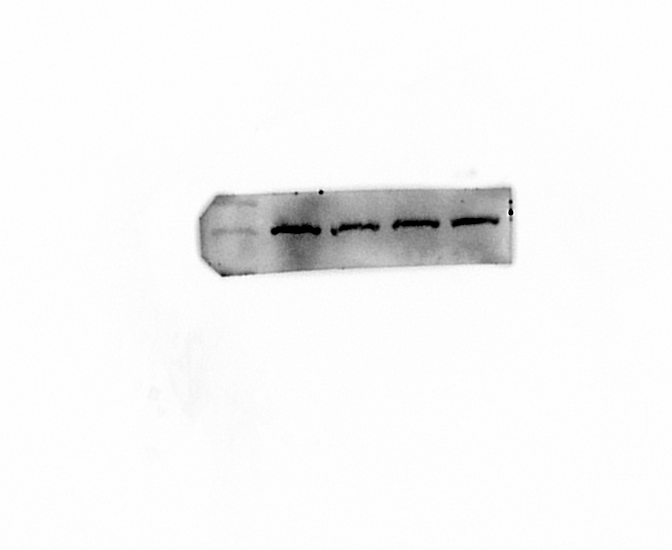

Supplement: Figure 7—source data 1. [file elife-94765-fig7-data1.zip › Figure 7-soure data 1/EGR1/GAPDH_2.tif]

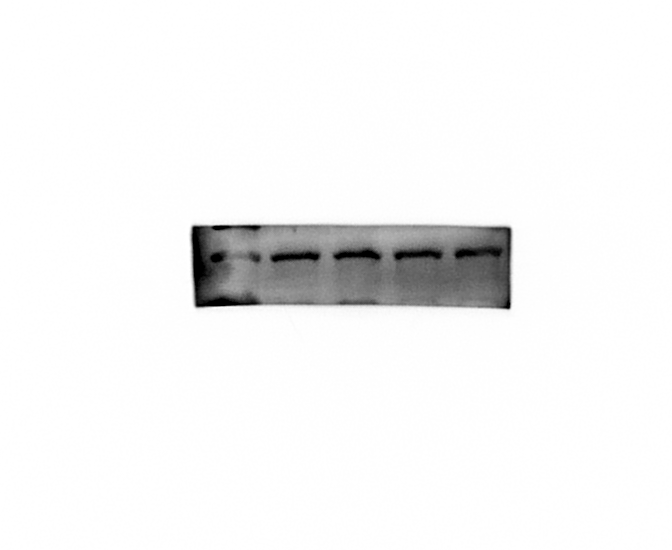

Supplement: Figure 7—source data 1. [file elife-94765-fig7-data1.zip › Figure 7-soure data 1/EGR1/GAPDH_1.tif]

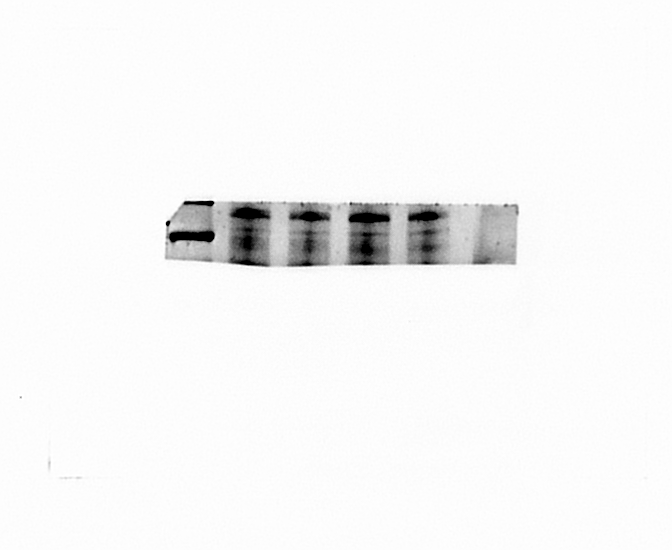

Supplement: Figure 7—source data 1. [file elife-94765-fig7-data1.zip › Figure 7-soure data 1/MEK/MEK-5.tif]

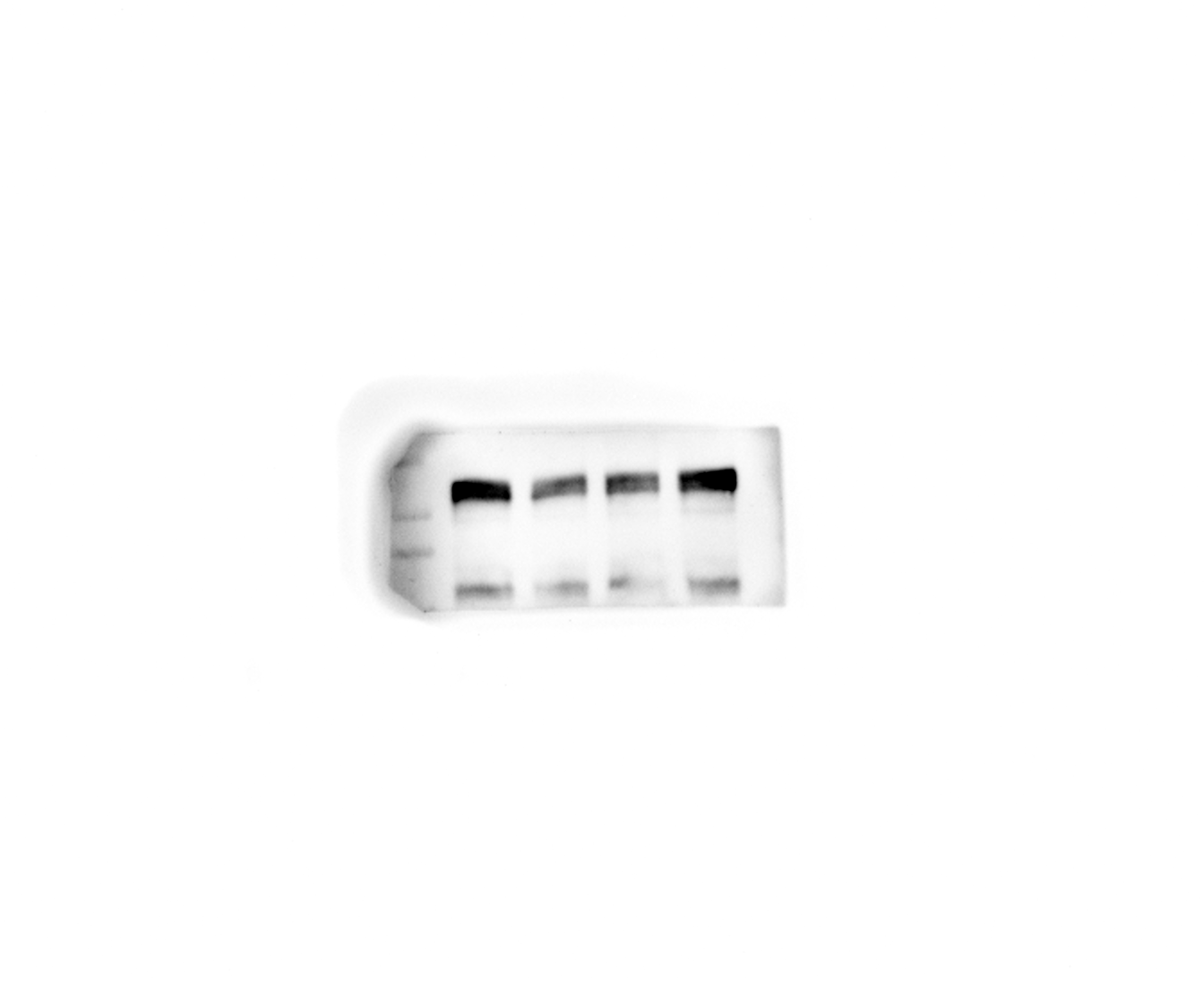

Supplement: Figure 7—source data 1. [file elife-94765-fig7-data1.zip › Figure 7-soure data 1/MEK/MEK-4.Tif]

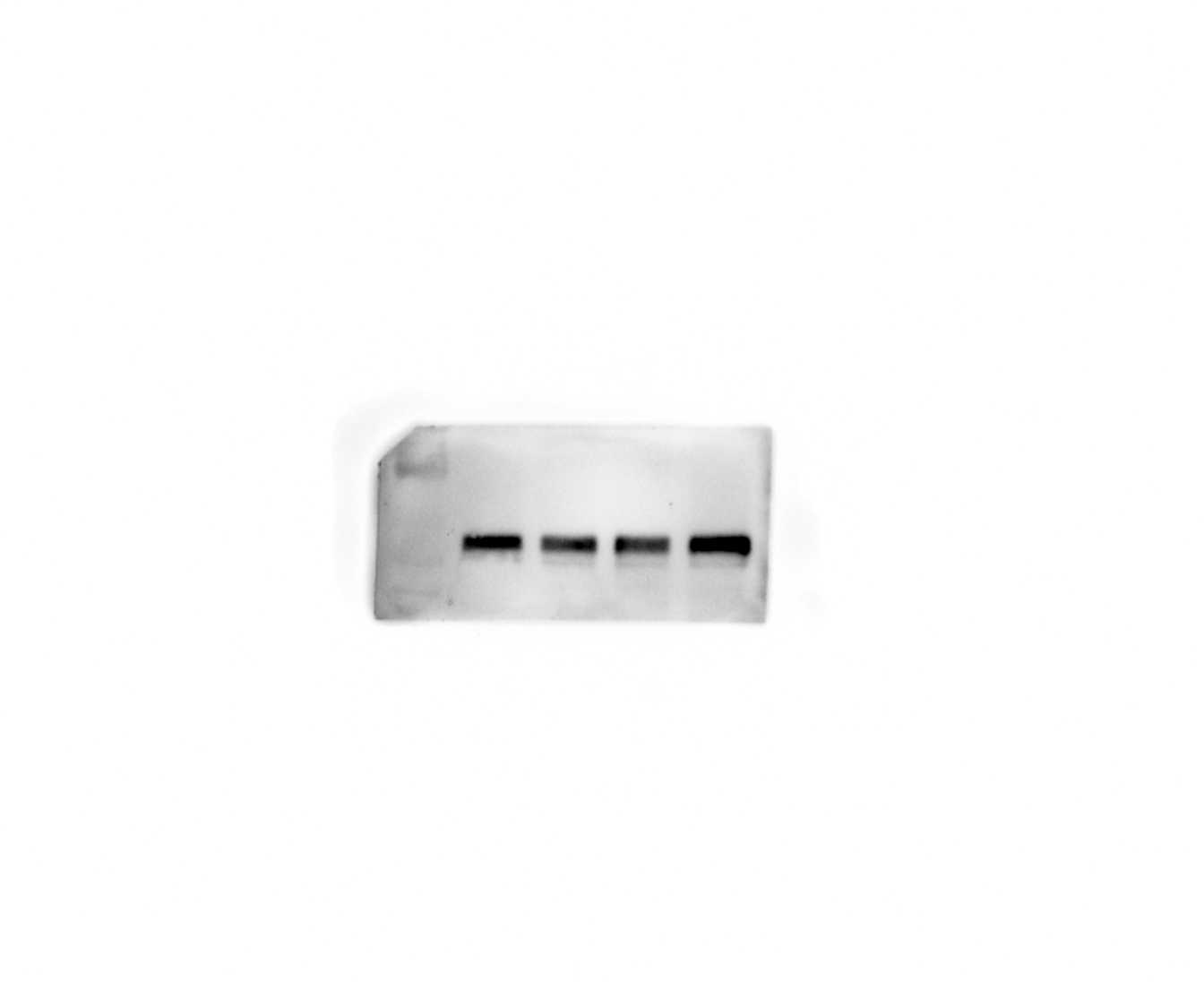

Supplement: Figure 7—source data 1. [file elife-94765-fig7-data1.zip › Figure 7-soure data 1/MEK/MEK-1.Tif]

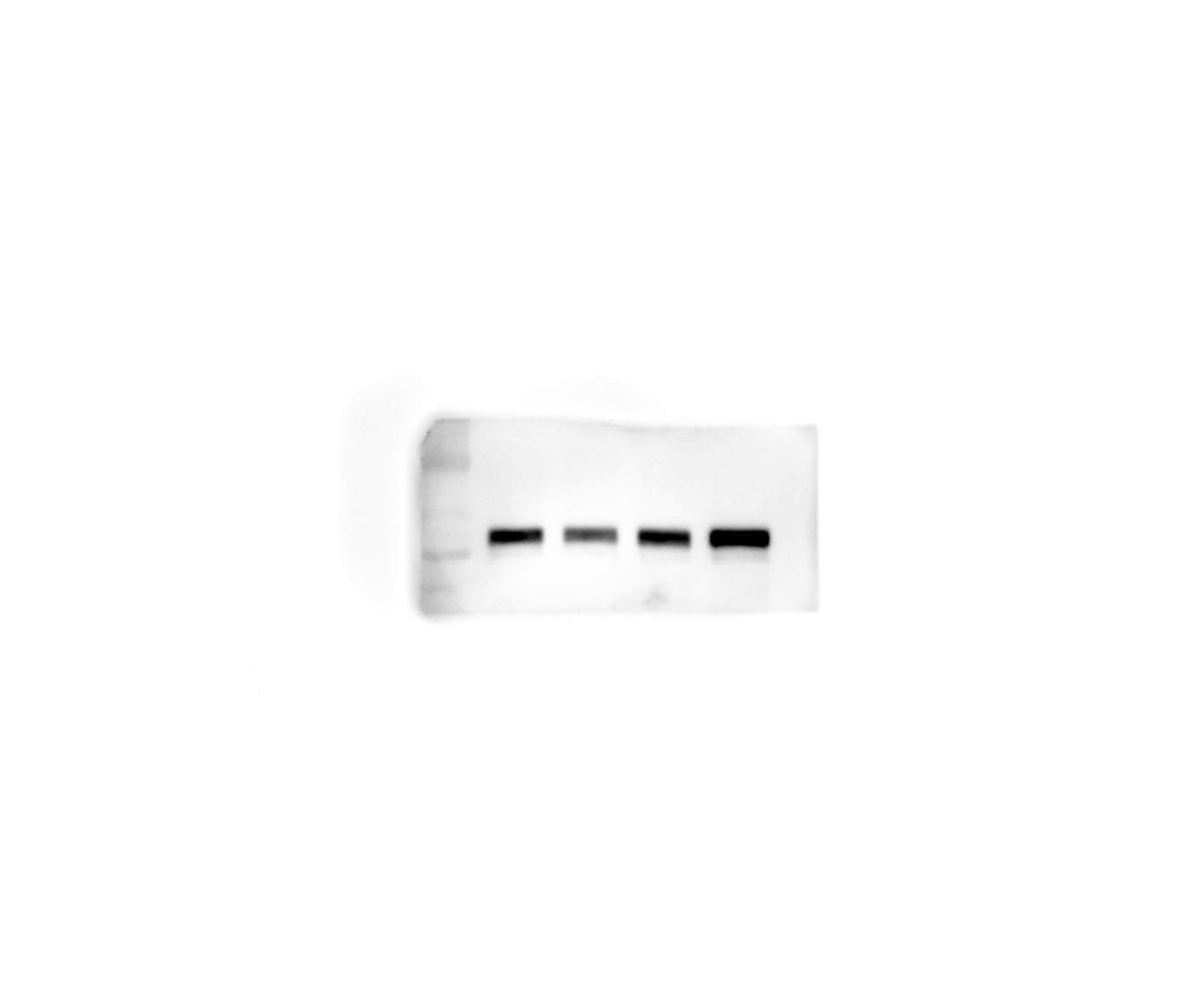

Supplement: Figure 7—source data 1. [file elife-94765-fig7-data1.zip › Figure 7-soure data 1/MEK/MEK-2.Tif]

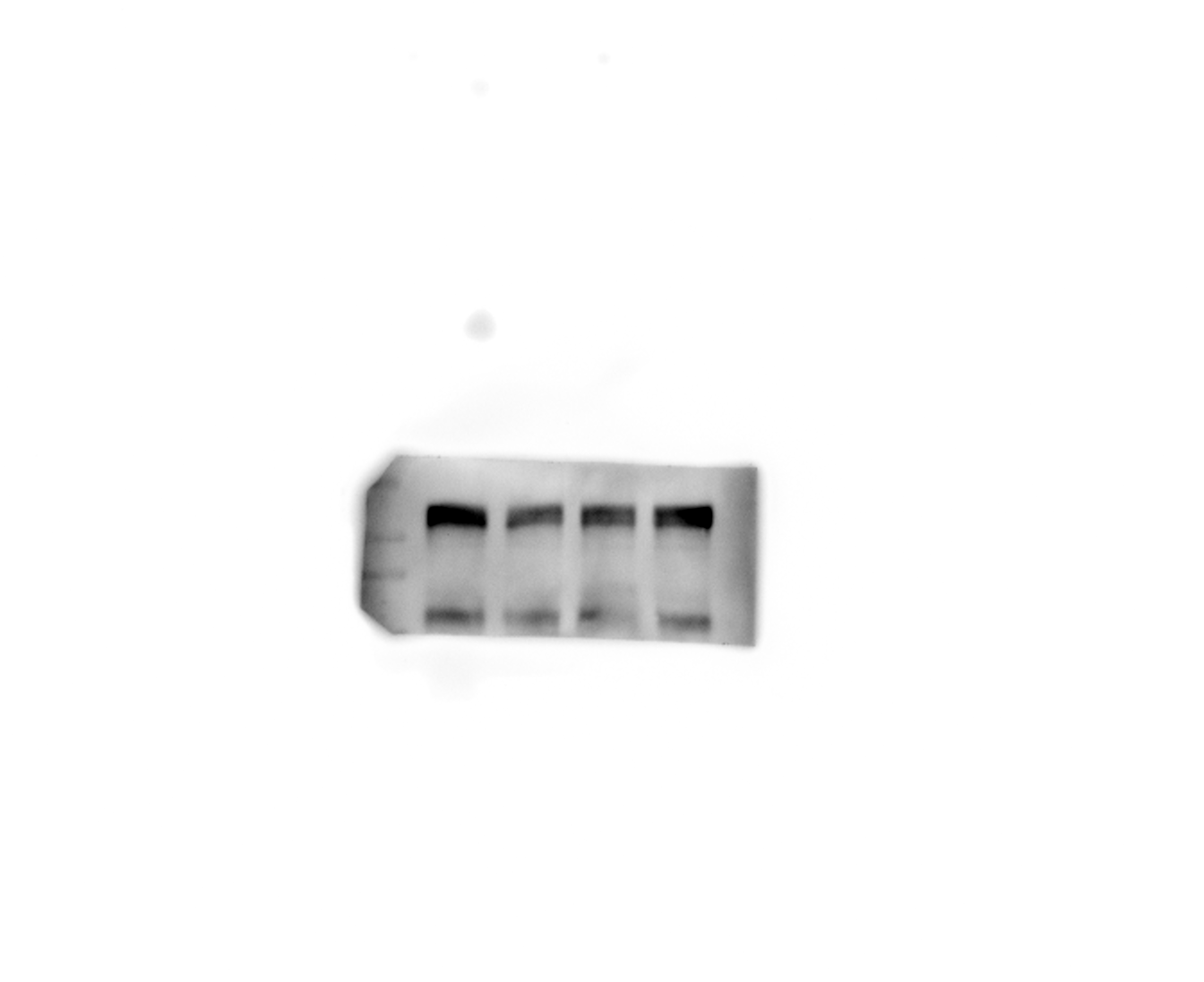

Supplement: Figure 7—source data 1. [file elife-94765-fig7-data1.zip › Figure 7-soure data 1/MEK/P-MEK-4.Tif]

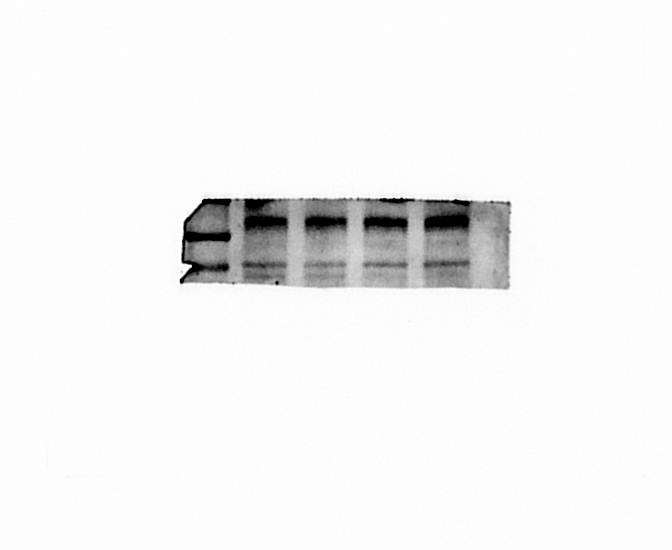

Supplement: Figure 7—source data 1. [file elife-94765-fig7-data1.zip › Figure 7-soure data 1/MEK/P-MEK-5.tif]

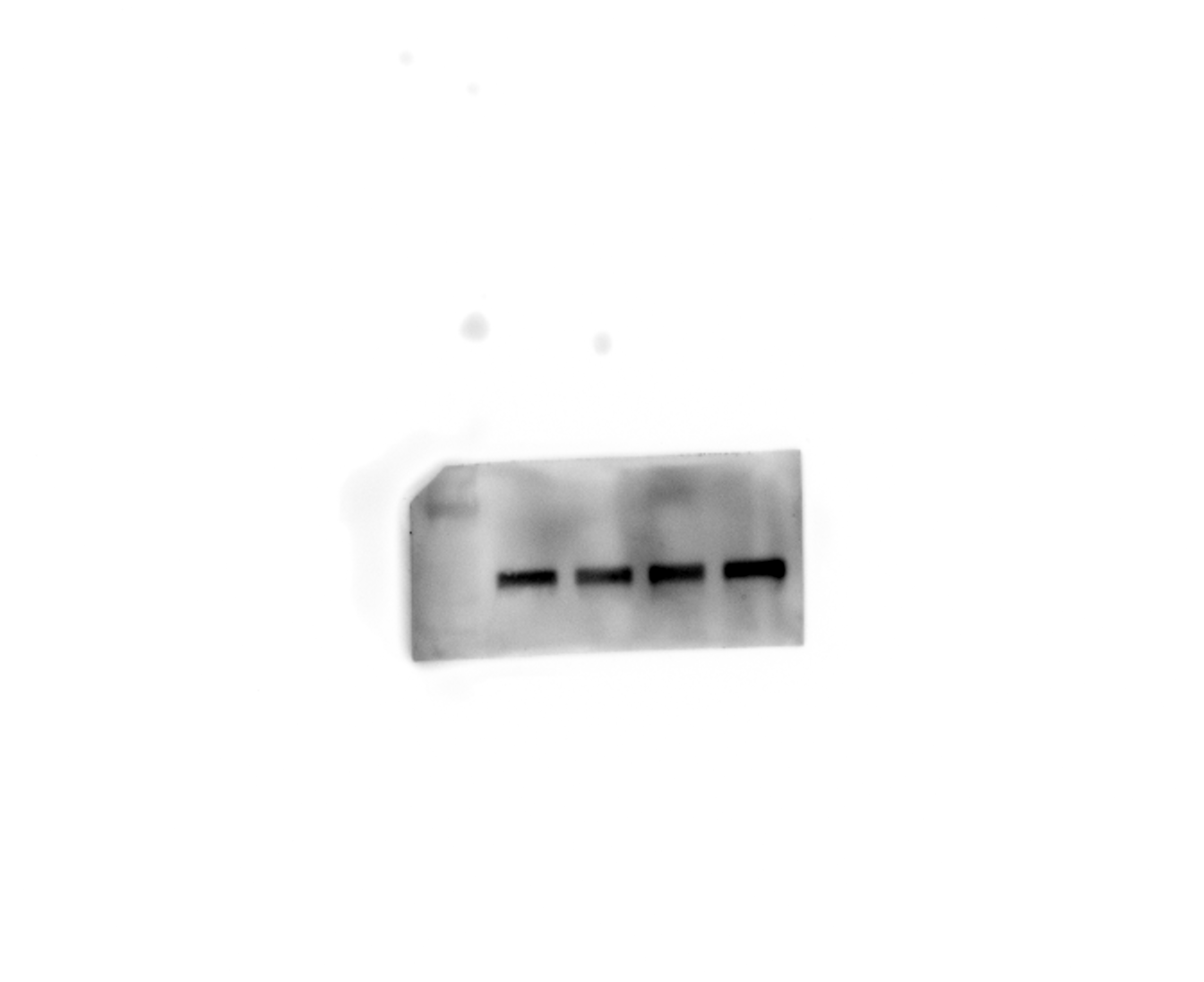

Supplement: Figure 7—source data 1. [file elife-94765-fig7-data1.zip › Figure 7-soure data 1/MEK/P-MEK-1.Tif]

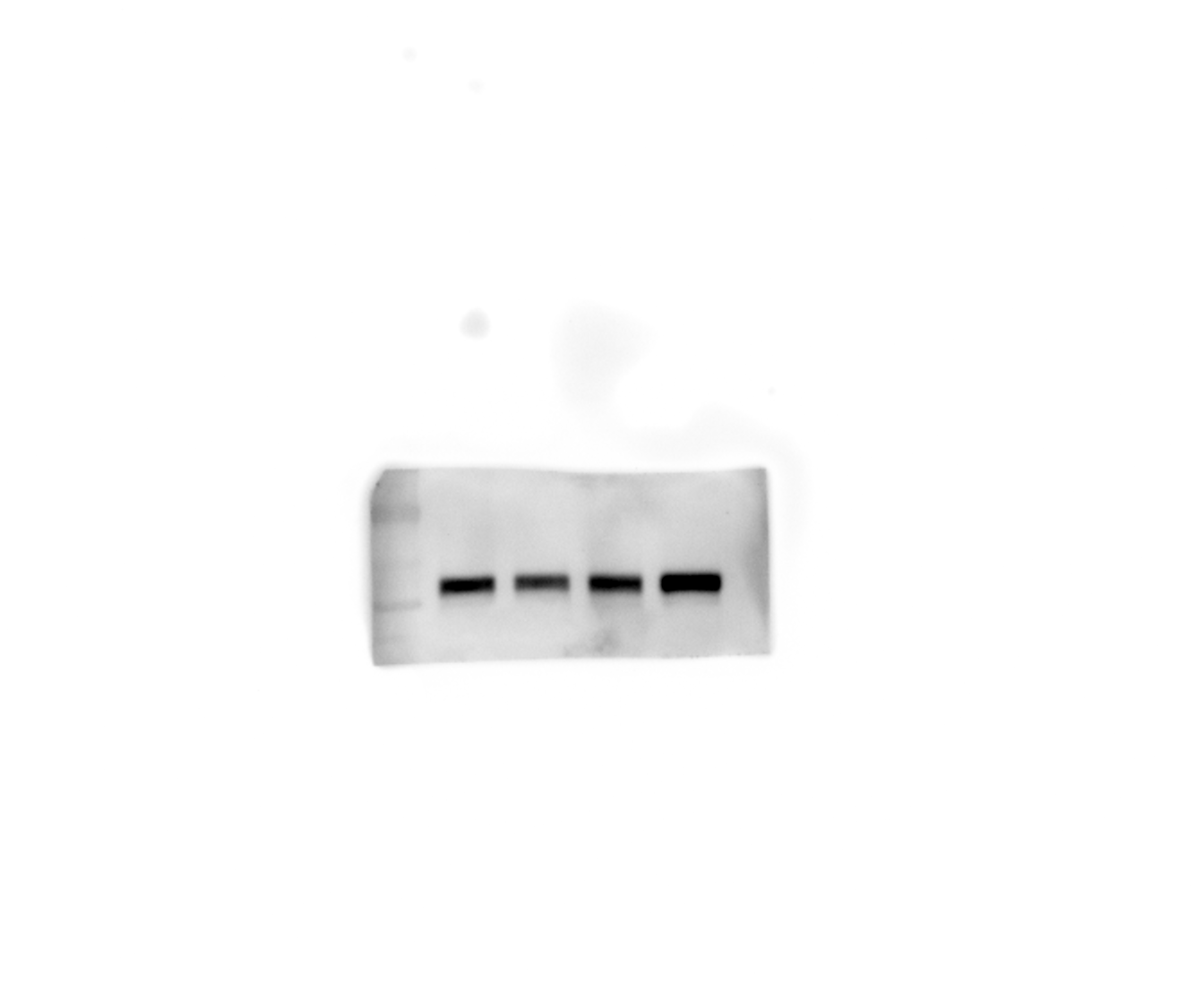

Supplement: Figure 7—source data 1. [file elife-94765-fig7-data1.zip › Figure 7-soure data 1/MEK/P-MEK-2.Tif]

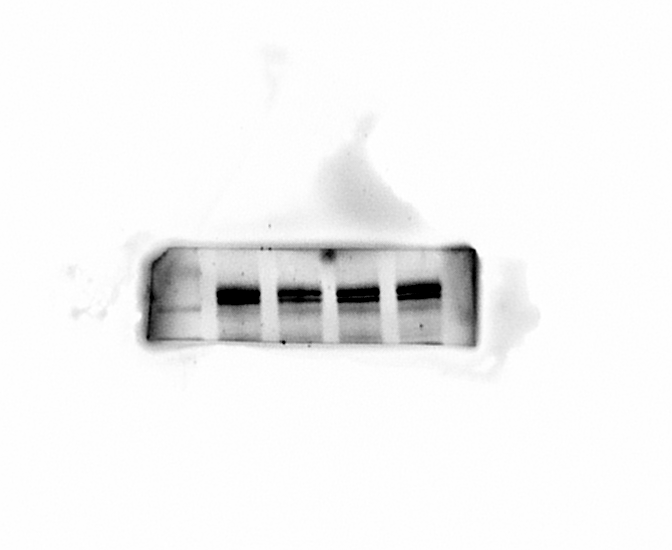

Supplement: Figure 7—source data 1. [file elife-94765-fig7-data1.zip › Figure 7-soure data 1/SRC/SRC_1.tif]

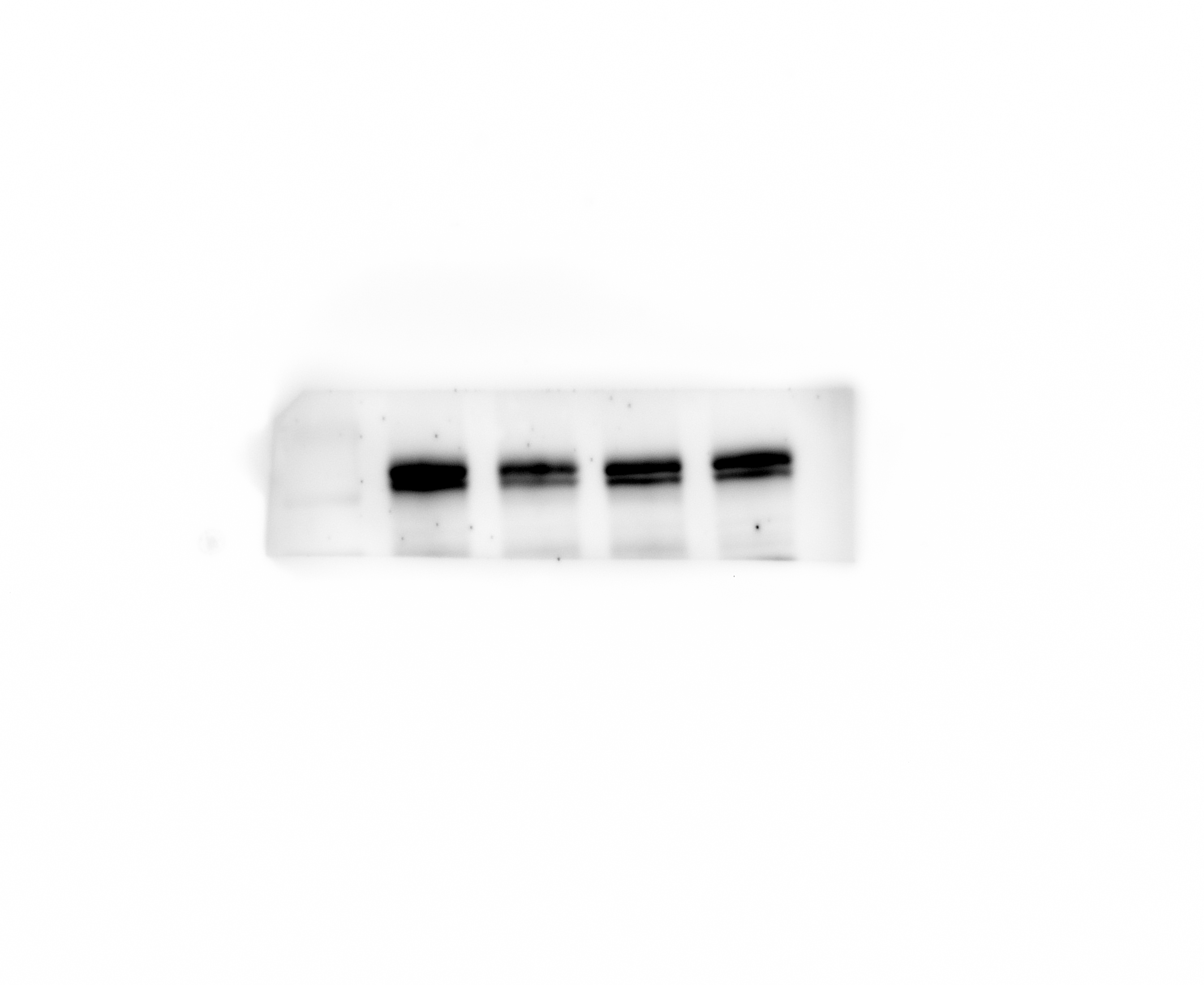

Supplement: Figure 7—source data 1. [file elife-94765-fig7-data1.zip › Figure 7-soure data 1/SRC/SRC_2.tif]

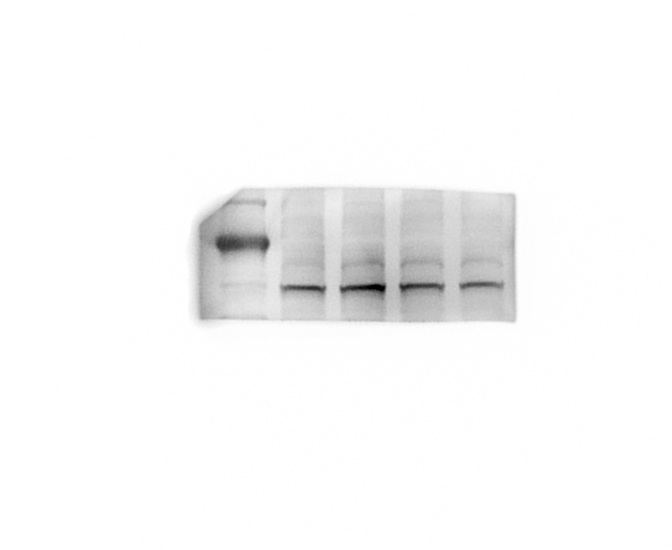

Supplement: Figure 7—source data 1. [file elife-94765-fig7-data1.zip › Figure 7-soure data 1/SRC/SRC_3.tif]

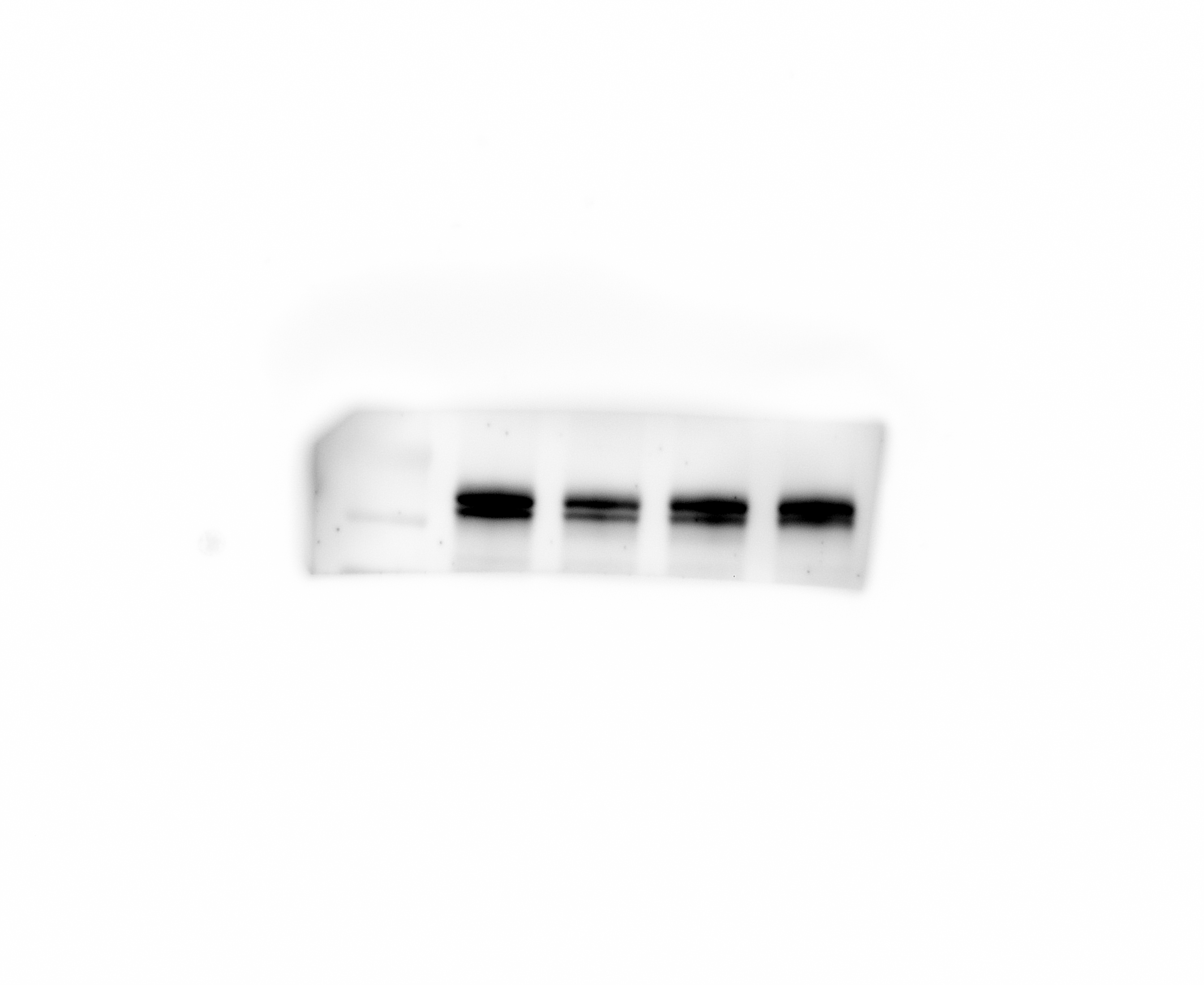

Supplement: Figure 7—source data 1. [file elife-94765-fig7-data1.zip › Figure 7-soure data 1/SRC/P_SRC_1.tif]

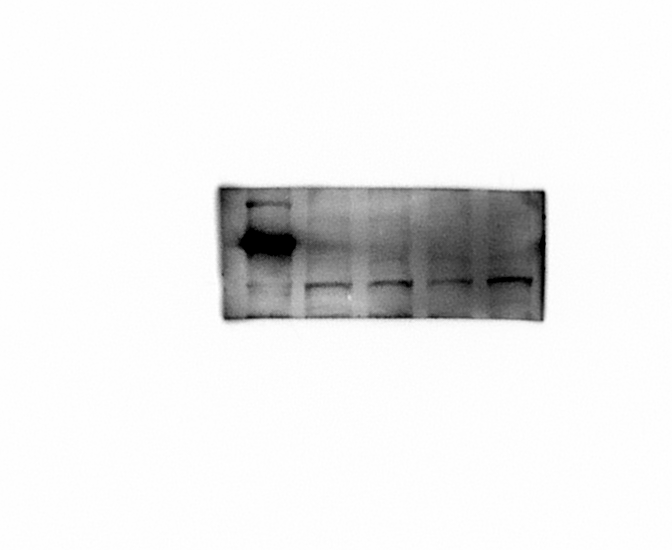

Supplement: Figure 7—source data 1. [file elife-94765-fig7-data1.zip › Figure 7-soure data 1/SRC/P_SRC_3.tif]

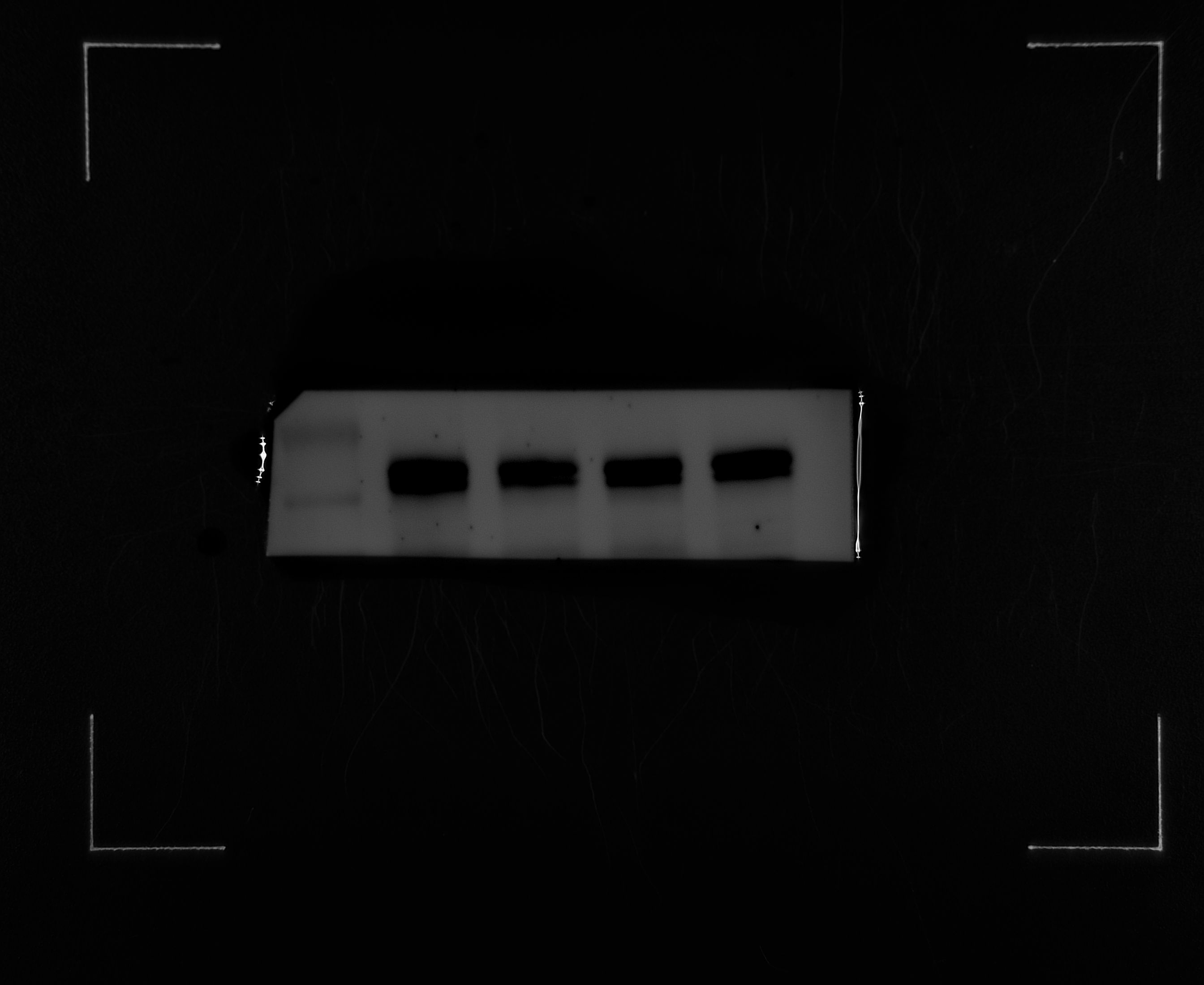

Supplement: Figure 7—source data 1. [file elife-94765-fig7-data1.zip › Figure 7-soure data 1/SRC/SRC_2_230208_142458_00.37.000_0_49053.tif]

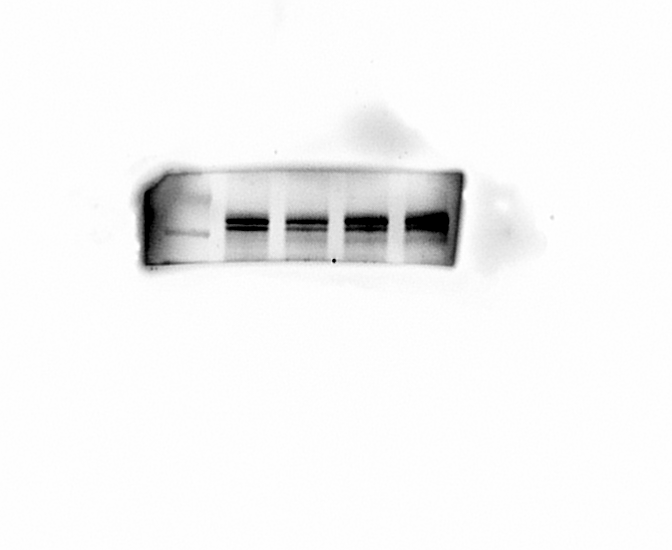

Supplement: Figure 7—source data 1. [file elife-94765-fig7-data1.zip › Figure 7-soure data 1/SRC/p-SRC_2.tif]

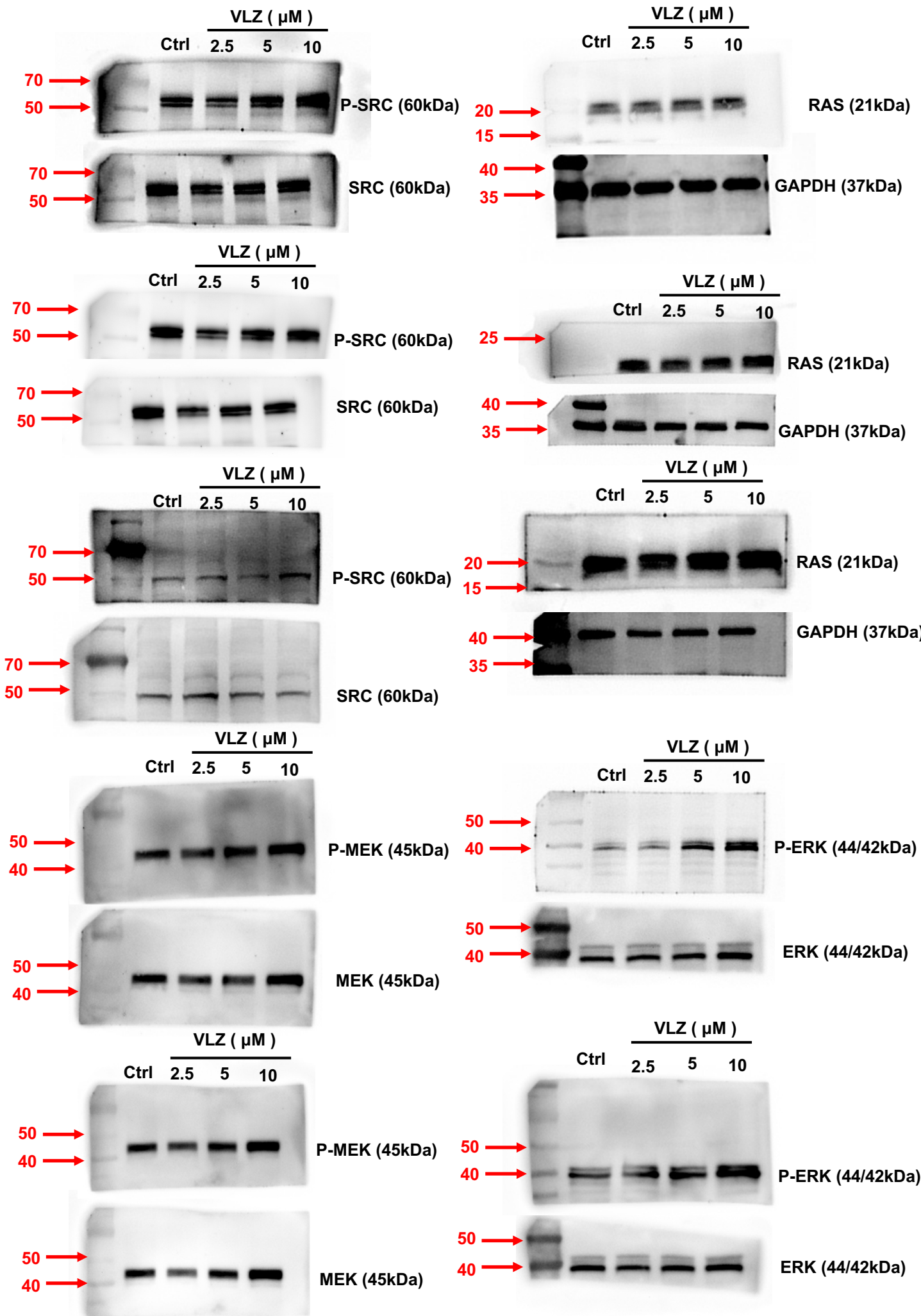

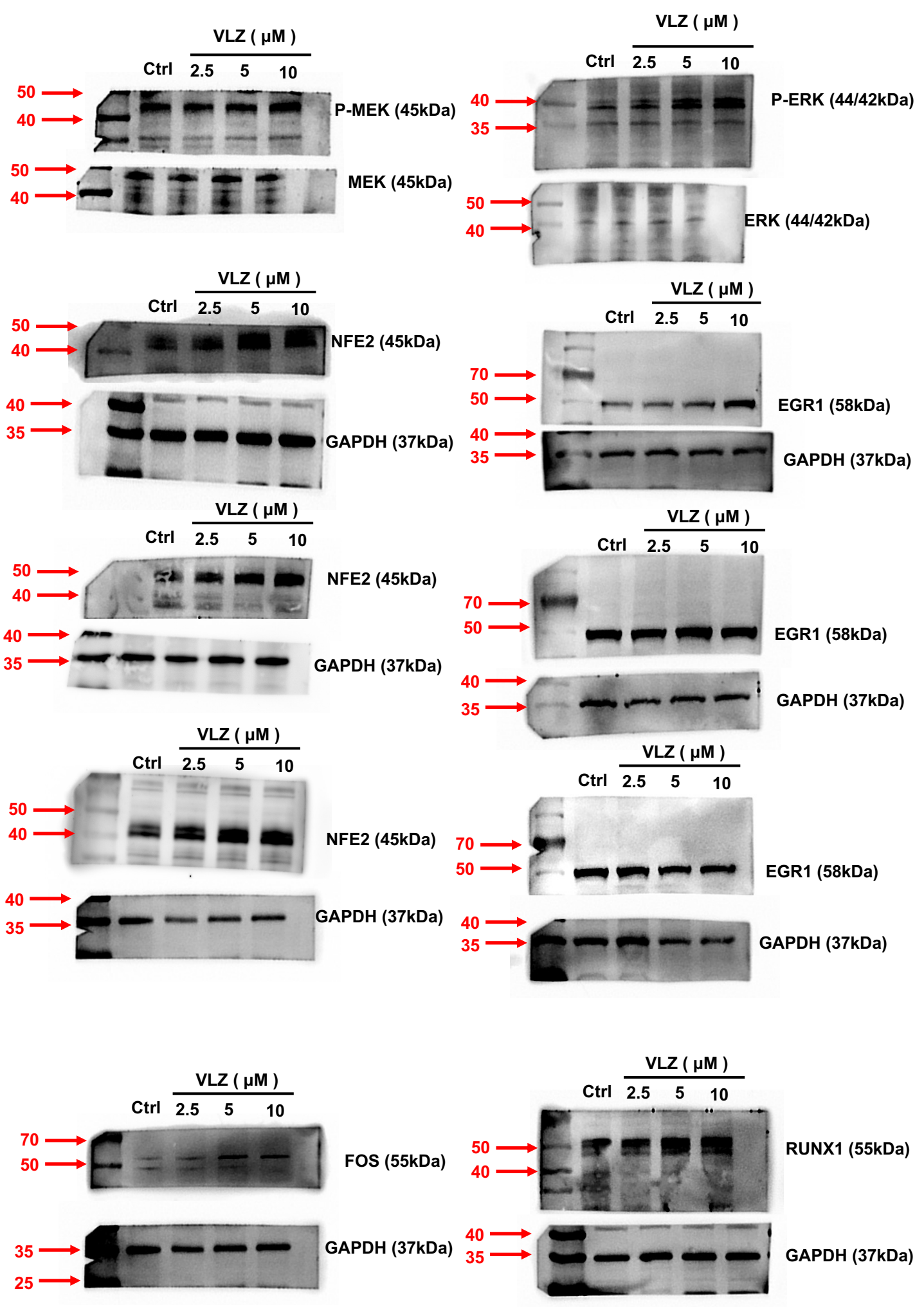

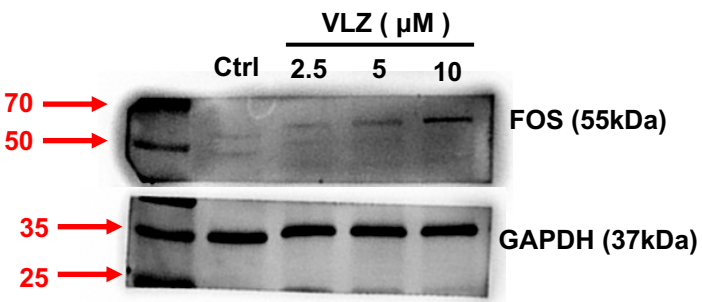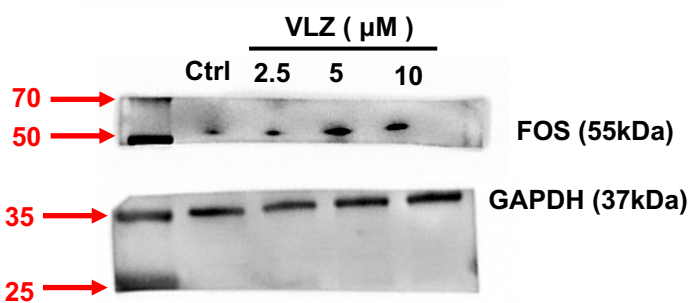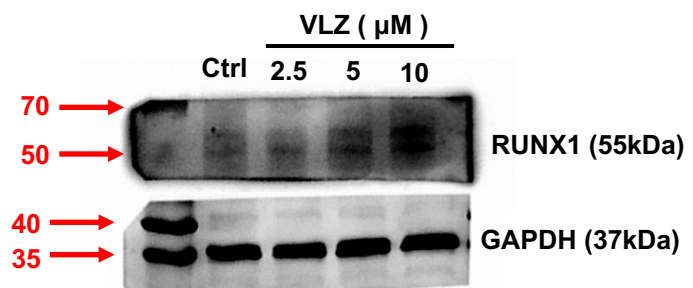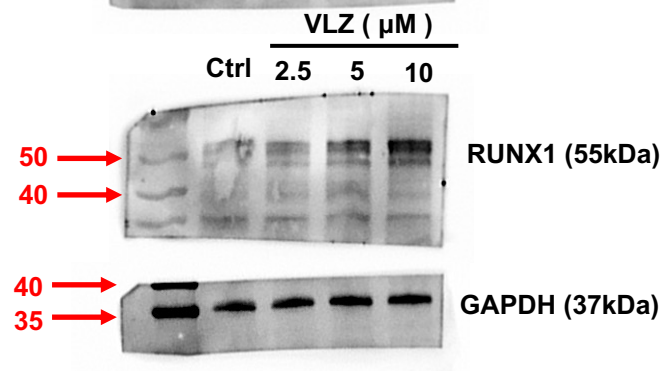

Supplement: Figure 7—source data 2. [file elife-94765-fig7-data2.pdf]

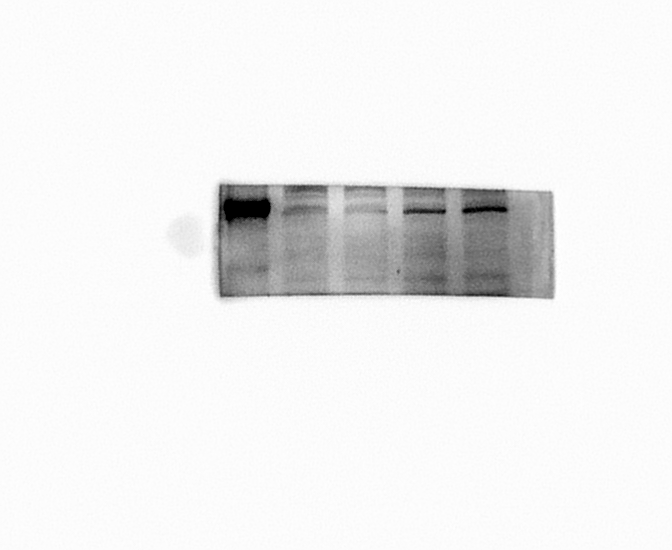

Supplement: Figure 8—source data 1. [file elife-94765-fig8-data1.zip › Figure 8-source data 1/5-HT1AR/5_HTR1A-1.tif]

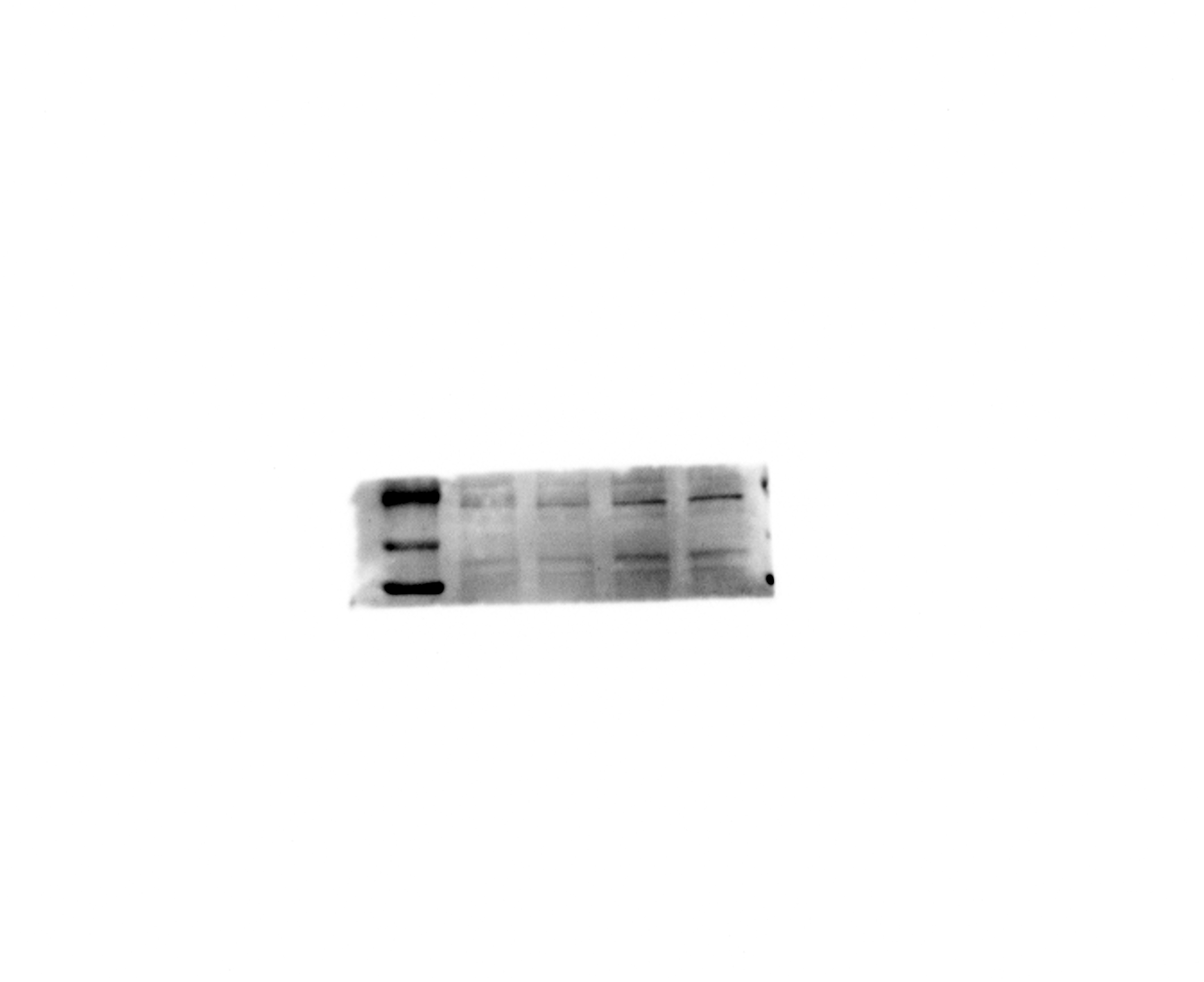

Supplement: Figure 8—source data 1. [file elife-94765-fig8-data1.zip › Figure 8-source data 1/5-HT1AR/5-HT1A-3.Tif]

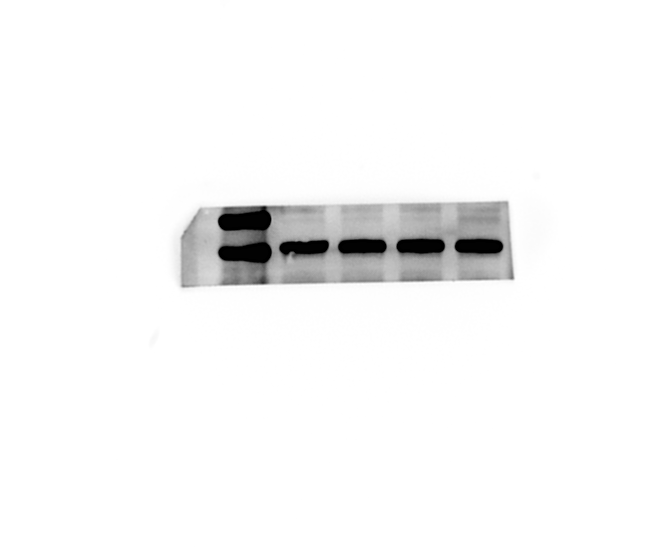

Supplement: Figure 8—source data 1. [file elife-94765-fig8-data1.zip › Figure 8-source data 1/5-HT1AR/GAPDH-1.tif]

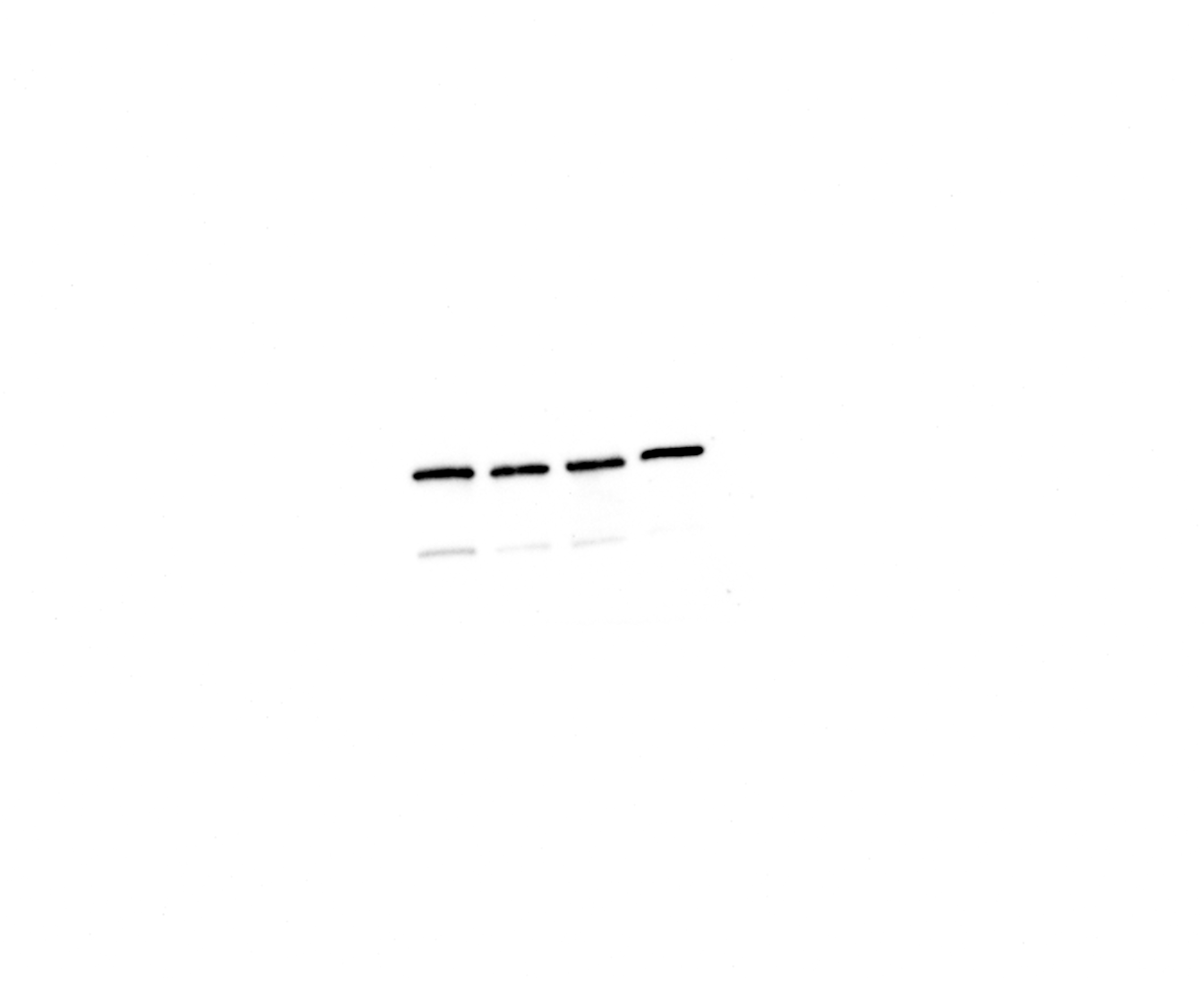

Supplement: Figure 8—source data 1. [file elife-94765-fig8-data1.zip › Figure 8-source data 1/5-HT1AR/GAPDH-3.Tif]

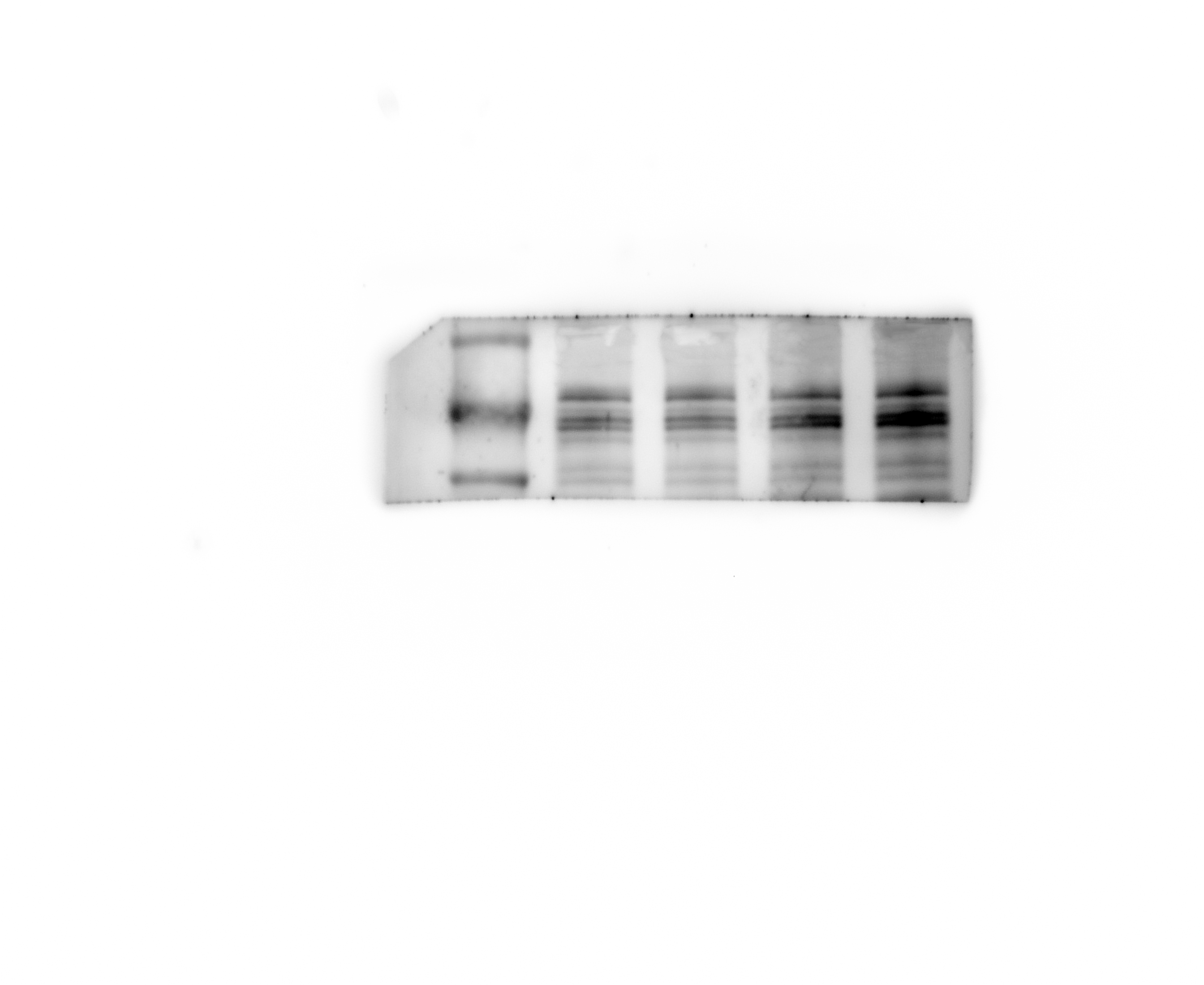

Supplement: Figure 8—source data 1. [file elife-94765-fig8-data1.zip › Figure 8-source data 1/5-HT1AR/5HT-2.tif]

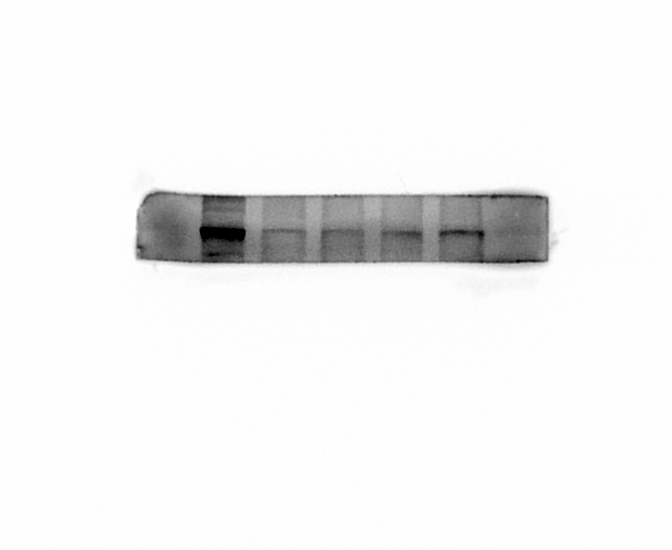

Supplement: Figure 8—source data 1. [file elife-94765-fig8-data1.zip › Figure 8-source data 1/Figure 8c/5_HT1A_3_230404_110800_00.04.000_1_15025.tif]

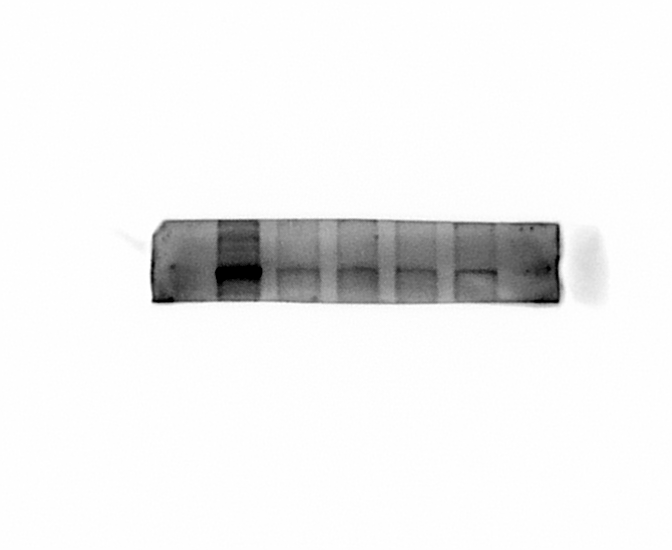

Supplement: Figure 8—source data 1. [file elife-94765-fig8-data1.zip › Figure 8-source data 1/Figure 8c/5_HT1A_1_230404_110355_00.04.000_1_15125.tif]

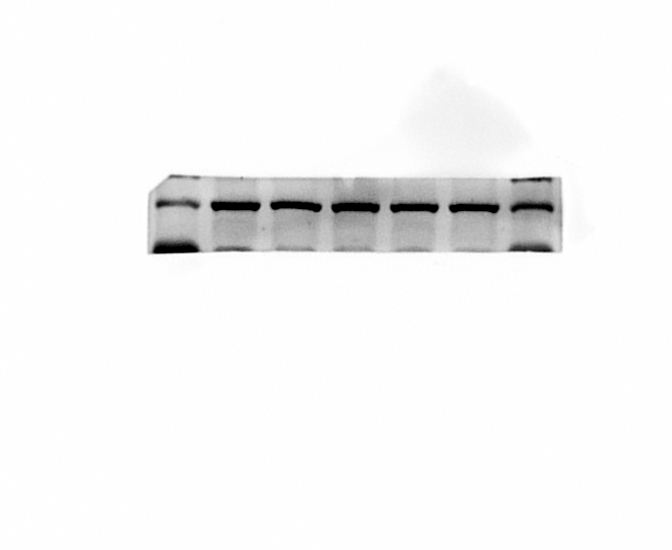

Supplement: Figure 8—source data 1. [file elife-94765-fig8-data1.zip › Figure 8-source data 1/Figure 8c/GAPDH_3_230404_110206_00.02.000_1_22062.tif]

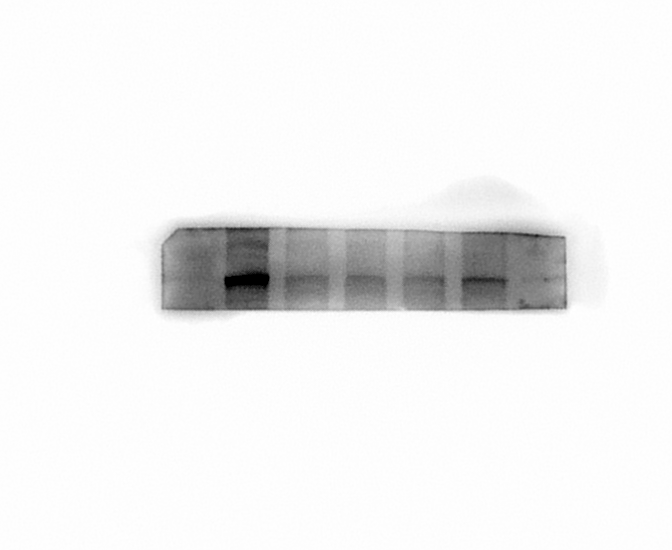

Supplement: Figure 8—source data 1. [file elife-94765-fig8-data1.zip › Figure 8-source data 1/Figure 8c/5_HT1A_2_230404_110553_00.03.000_1_14128.tif]

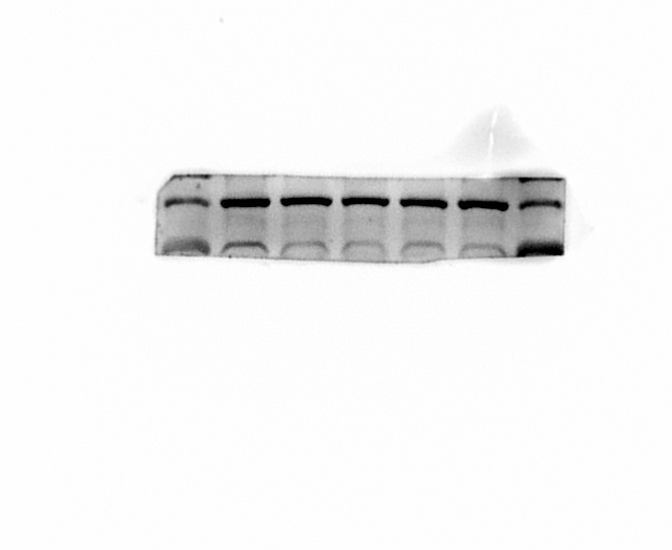

Supplement: Figure 8—source data 1. [file elife-94765-fig8-data1.zip › Figure 8-source data 1/Figure 8c/GAPDH_2_230404_110036_00.02.000_1_14266.tif]

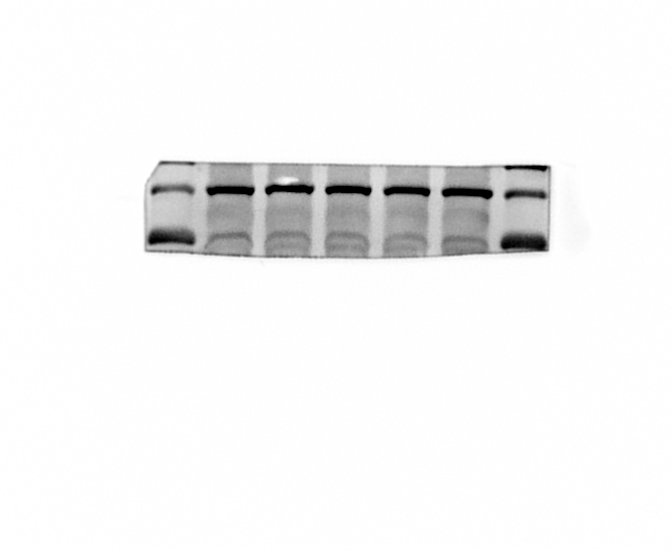

Supplement: Figure 8—source data 1. [file elife-94765-fig8-data1.zip › Figure 8-source data 1/Figure 8c/GAPDH_1_230404_105919_00.02.000_1_24999.tif]

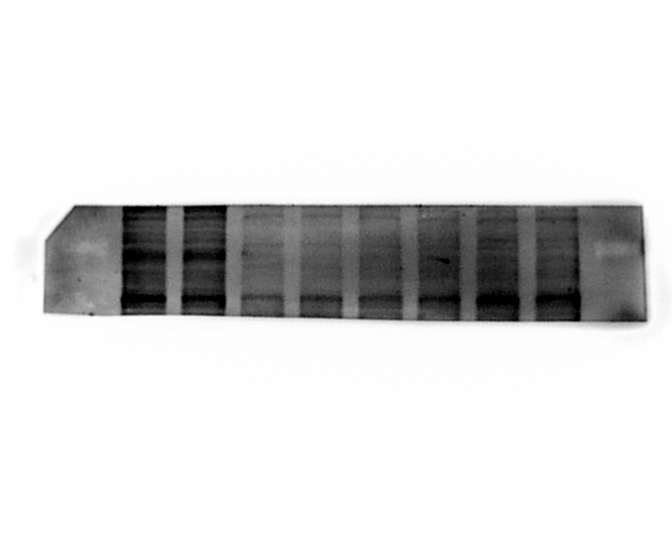

Supplement: Figure 8—source data 1. [file elife-94765-fig8-data1.zip › Figure 8-source data 1/figure 8b/5_HT1A_4_230402_112601_00.11.000_2_26950.tif]

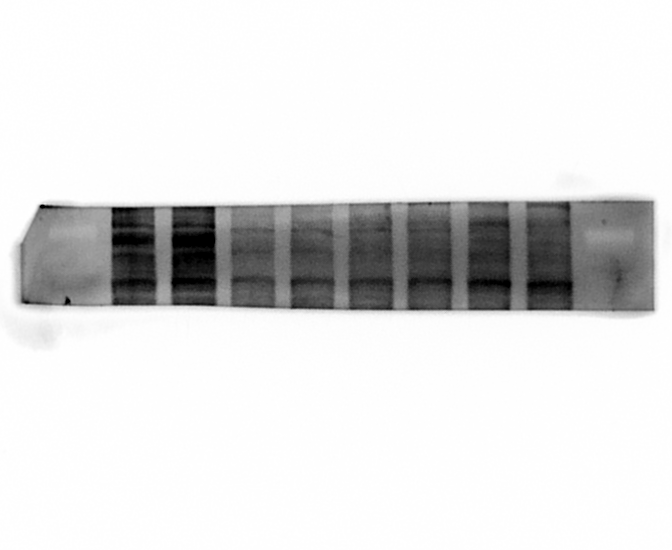

Supplement: Figure 8—source data 1. [file elife-94765-fig8-data1.zip › Figure 8-source data 1/figure 8b/5_HT1A_2_230402_111939_00.10.000_1_27988.tif]

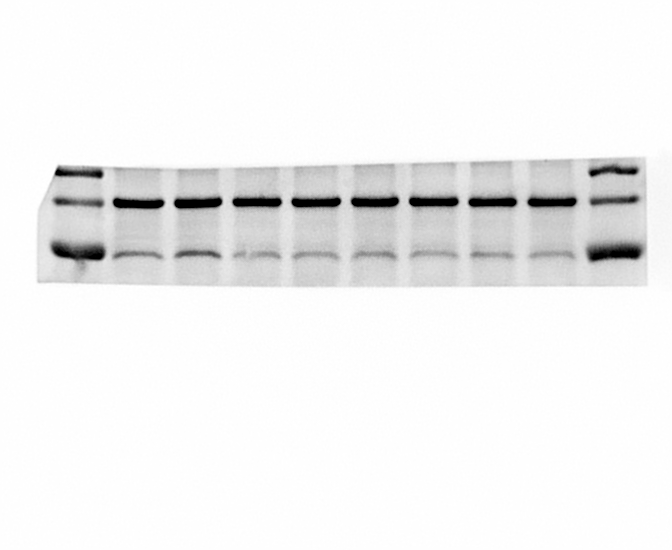

Supplement: Figure 8—source data 1. [file elife-94765-fig8-data1.zip › Figure 8-source data 1/figure 8b/GAPDH_2_230402_110949_00.01.000_1_18600.tif]

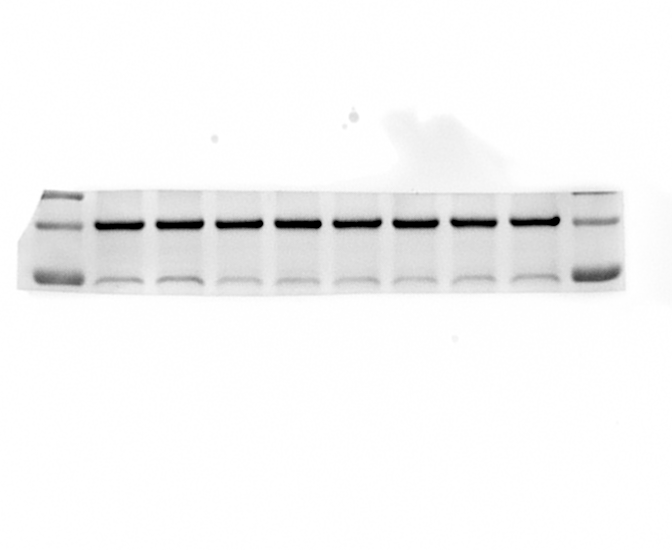

Supplement: Figure 8—source data 1. [file elife-94765-fig8-data1.zip › Figure 8-source data 1/figure 8b/GAPDH_4_230402_111341_00.02.000_1_29416.tif]

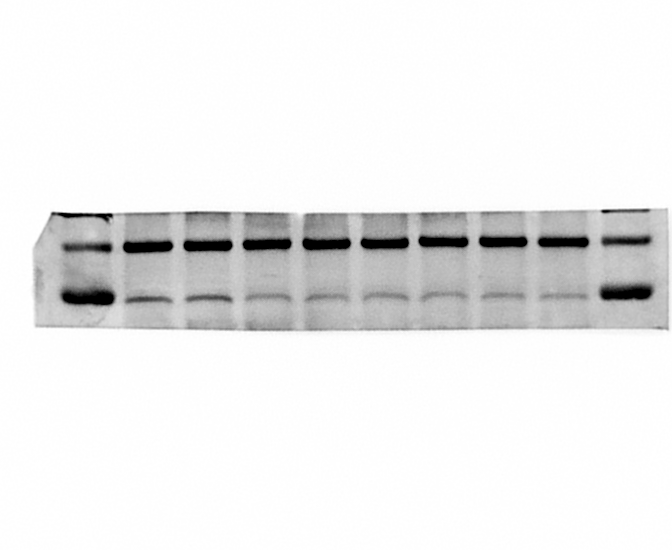

Supplement: Figure 8—source data 1. [file elife-94765-fig8-data1.zip › Figure 8-source data 1/figure 8b/GAPDH_1_230402_110737_00.01.000_1_21093.tif]

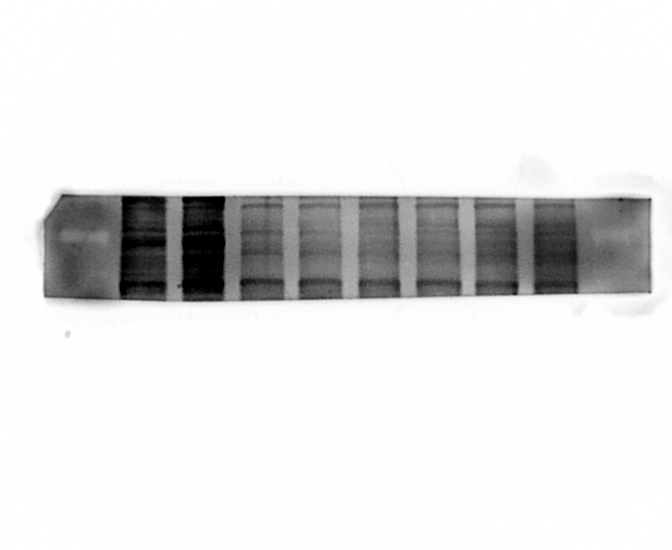

Supplement: Figure 8—source data 1. [file elife-94765-fig8-data1.zip › Figure 8-source data 1/figure 8b/5_HT1A_1_230402_111626_00.09.000_2_26540.tif]

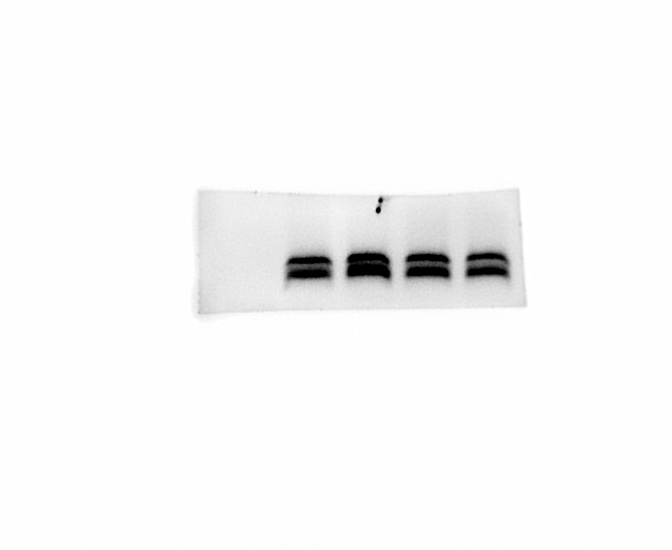

Supplement: Figure 8—source data 1. [file elife-94765-fig8-data1.zip › Figure 8-source data 1/Figure 8l/RAS/RAS_1_230331_162430_00.03.000_2_19660.tif]

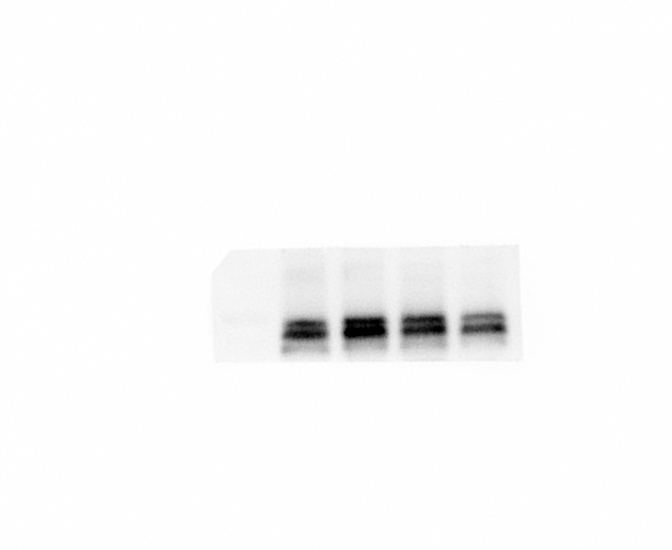

Supplement: Figure 8—source data 1. [file elife-94765-fig8-data1.zip › Figure 8-source data 1/Figure 8l/RAS/RAS_5_230509_114920_00.02.000_1_22098.tif]

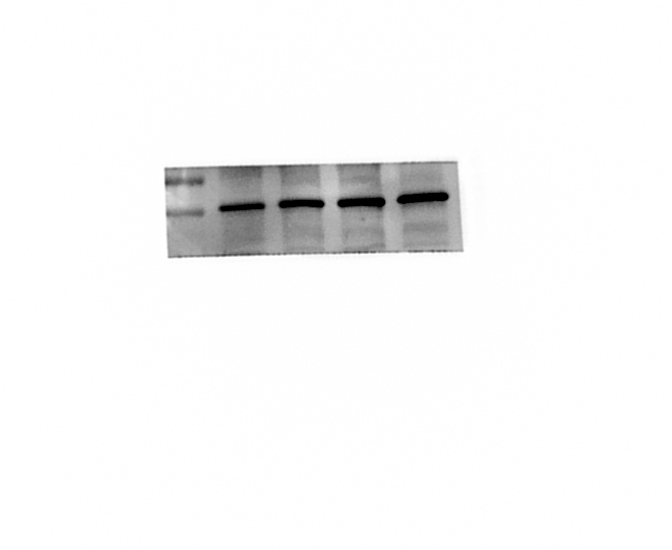

Supplement: Figure 8—source data 1. [file elife-94765-fig8-data1.zip › Figure 8-source data 1/Figure 8l/RAS/GAPDH_5_230509_111011_00.02.000_1_31470.tif]

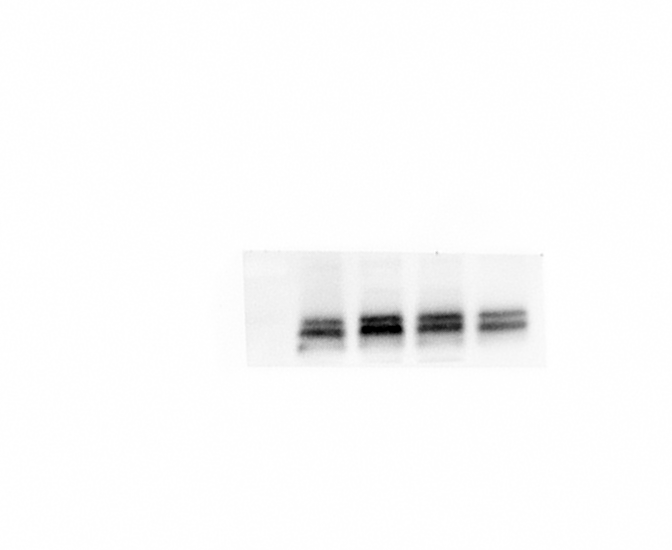

Supplement: Figure 8—source data 1. [file elife-94765-fig8-data1.zip › Figure 8-source data 1/Figure 8l/RAS/RAS_6_230509_115040_00.02.000_1_24514.tif]

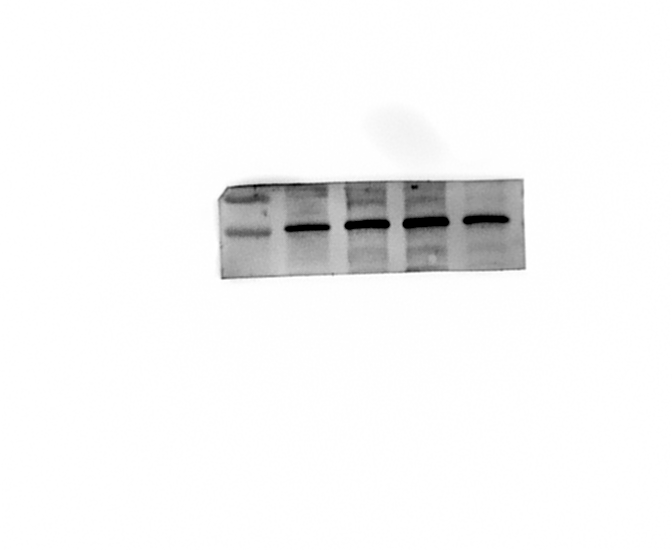

Supplement: Figure 8—source data 1. [file elife-94765-fig8-data1.zip › Figure 8-source data 1/Figure 8l/RAS/GAPDH_6_230509_111157_00.02.000_1_32378.tif]

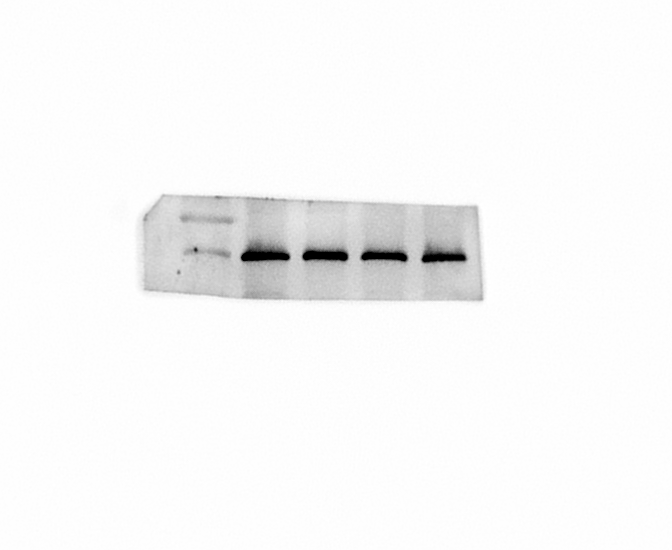

Supplement: Figure 8—source data 1. [file elife-94765-fig8-data1.zip › Figure 8-source data 1/Figure 8l/RAS/GAPDH_1_230331_161101_00.02.000_1_13059.tif]

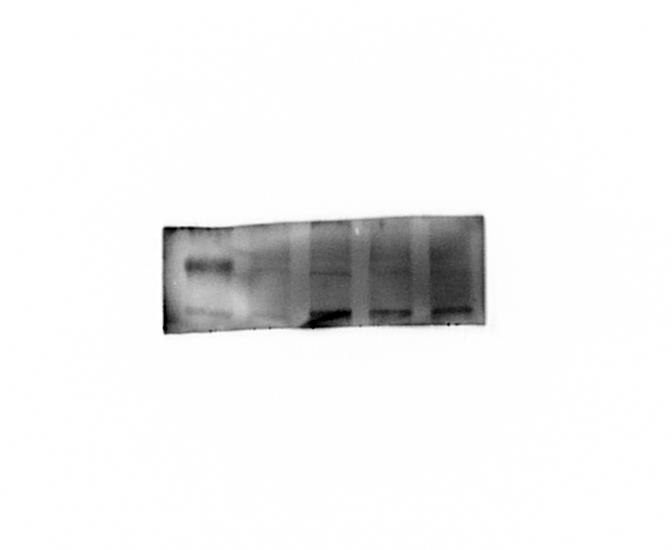

Supplement: Figure 8—source data 1. [file elife-94765-fig8-data1.zip › Figure 8-source data 1/Figure 8l/5-HT1AR/5_HT1A-1.tif]

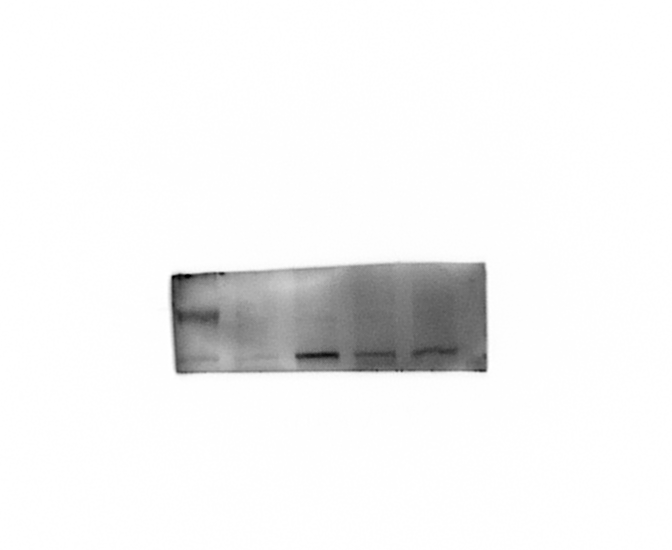

Supplement: Figure 8—source data 1. [file elife-94765-fig8-data1.zip › Figure 8-source data 1/Figure 8l/5-HT1AR/5_HT1A-2.tif]

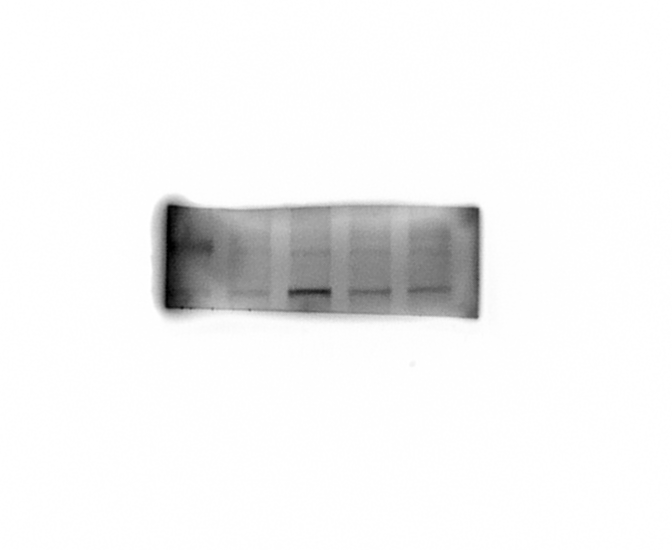

Supplement: Figure 8—source data 1. [file elife-94765-fig8-data1.zip › Figure 8-source data 1/Figure 8l/5-HT1AR/5_HT1A_3.tif]

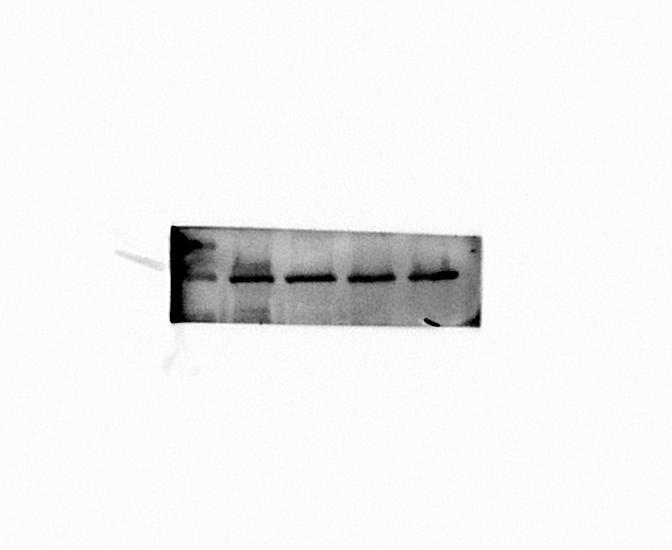

Supplement: Figure 8—source data 1. [file elife-94765-fig8-data1.zip › Figure 8-source data 1/Figure 8l/5-HT1AR/GAPDH_3_230325_161837_00.02.000_1_7478.tif]

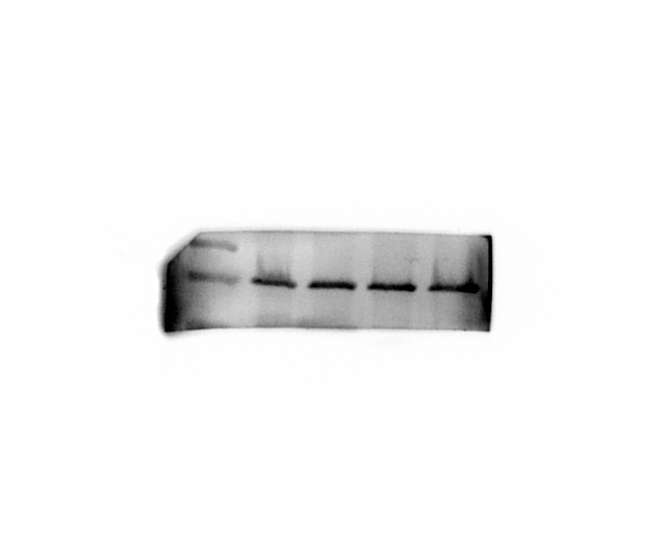

Supplement: Figure 8—source data 1. [file elife-94765-fig8-data1.zip › Figure 8-source data 1/Figure 8l/5-HT1AR/GAPDH_2_230325_162002_00.04.000_1_25613.tif]

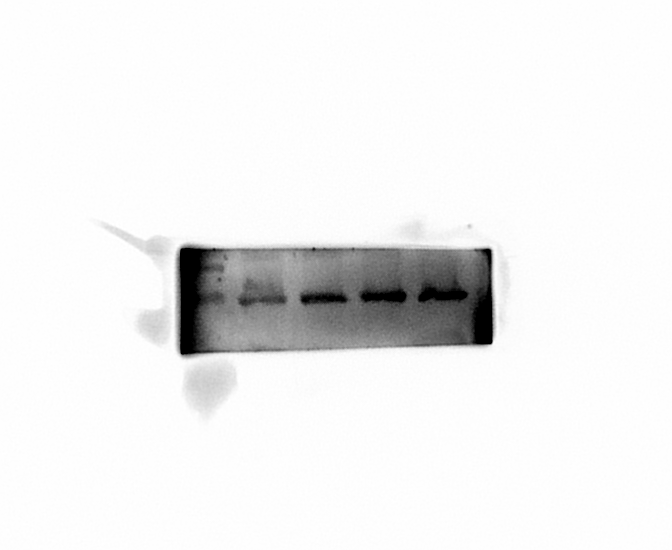

Supplement: Figure 8—source data 1. [file elife-94765-fig8-data1.zip › Figure 8-source data 1/Figure 8l/5-HT1AR/GAPDH_1_230325_161648_00.04.000_1_12508.tif]

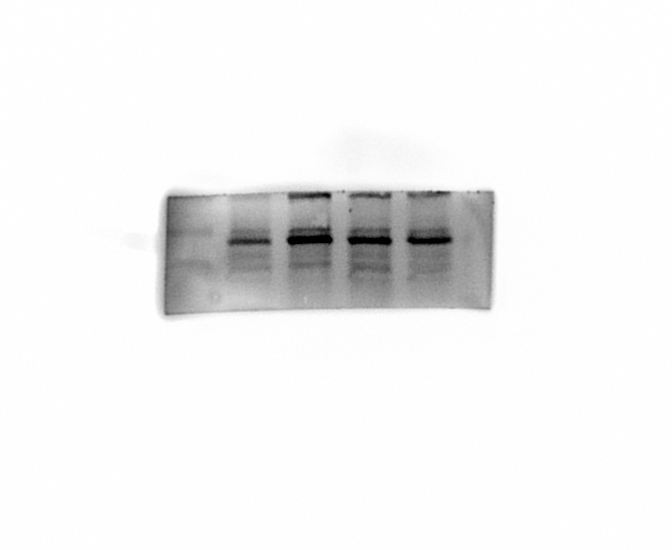

Supplement: Figure 8—source data 1. [file elife-94765-fig8-data1.zip › Figure 8-source data 1/Figure 8l/ERK/P_ERK_2_230325_164323_00.07.000_1_20020.tif]

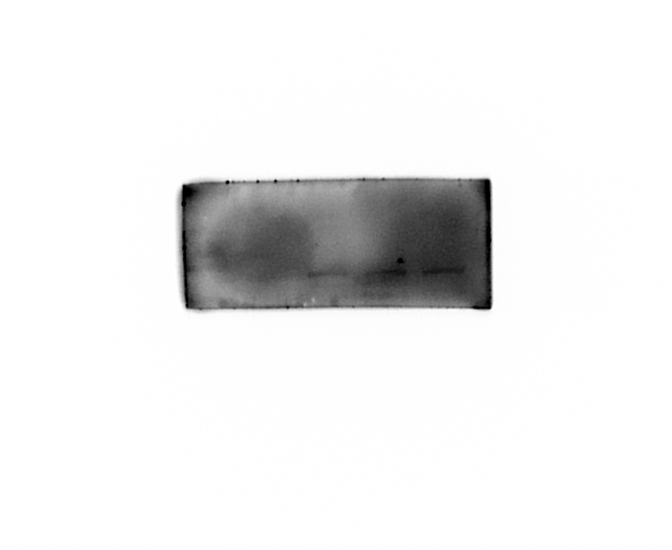

Supplement: Figure 8—source data 1. [file elife-94765-fig8-data1.zip › Figure 8-source data 1/Figure 8l/ERK/ERK_5_230401_111134_00.13.000_2_30820.tif]

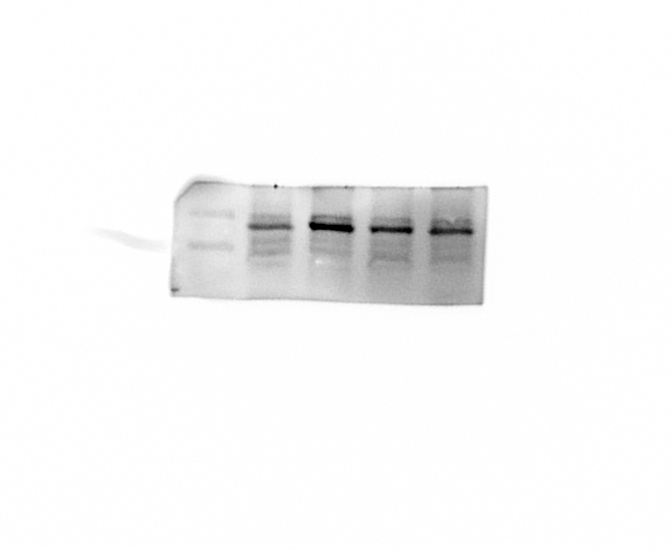

Supplement: Figure 8—source data 1. [file elife-94765-fig8-data1.zip › Figure 8-source data 1/Figure 8l/ERK/P_ERK_1_230325_164109_00.07.000_2_20782.tif]

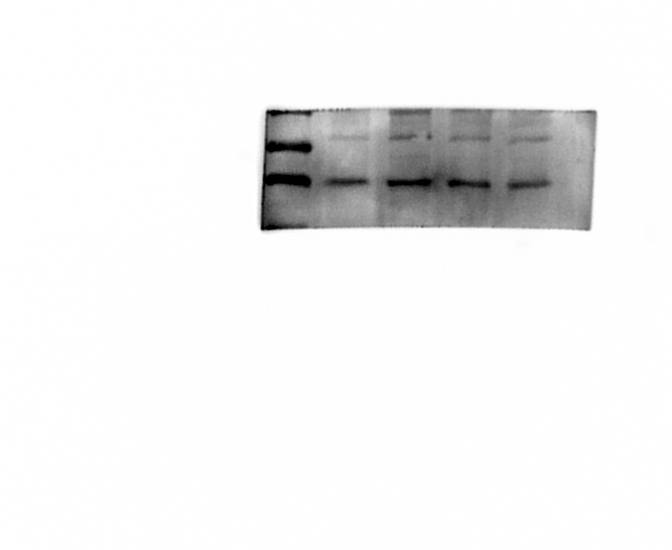

Supplement: Figure 8—source data 1. [file elife-94765-fig8-data1.zip › Figure 8-source data 1/Figure 8l/ERK/ERK_2_230328_113607_00.08.000_2_23296.tif]

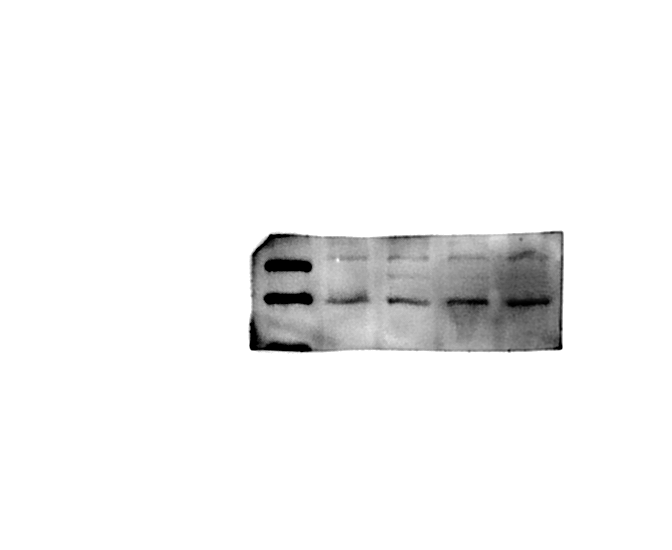

Supplement: Figure 8—source data 1. [file elife-94765-fig8-data1.zip › Figure 8-source data 1/Figure 8l/ERK/ERK_1_230328_113417_00.09.000_1_16231.tif]

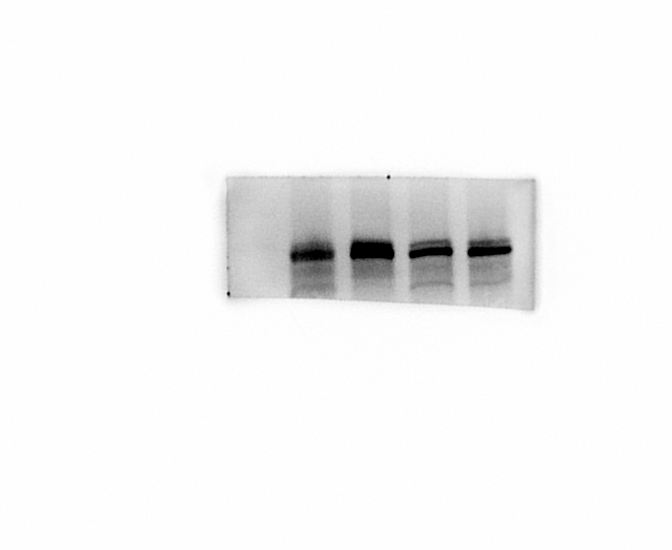

Supplement: Figure 8—source data 1. [file elife-94765-fig8-data1.zip › Figure 8-source data 1/Figure 8l/ERK/P_ERK_5_230331_162114_00.04.000_1_15875.tif]

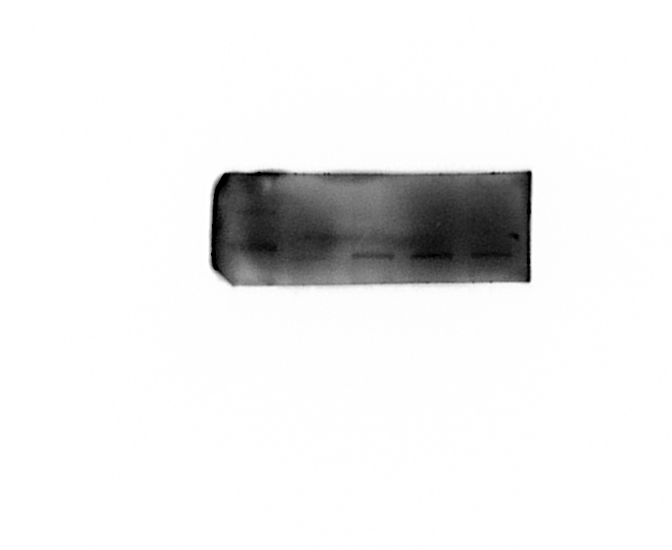

Supplement: Figure 8—source data 1. [file elife-94765-fig8-data1.zip › Figure 8-source data 1/Figure 8l/ERK/ERK_4_230401_110840_00.14.000_1_25132.tif]

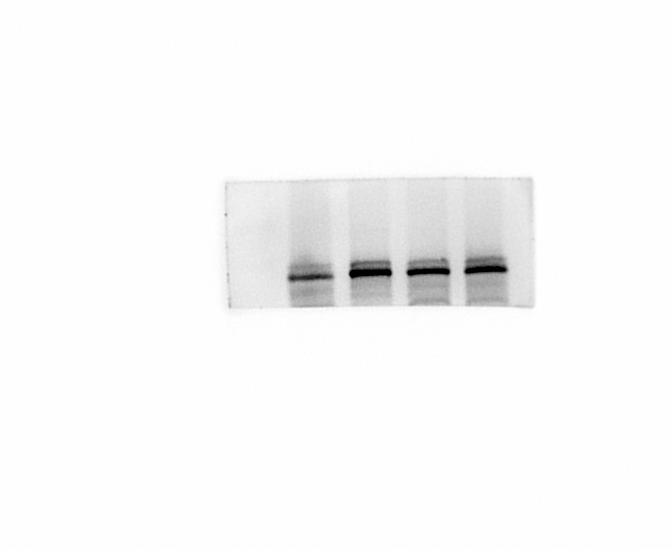

Supplement: Figure 8—source data 1. [file elife-94765-fig8-data1.zip › Figure 8-source data 1/Figure 8l/ERK/P_ERK_4_230331_161930_00.03.000_1_18163.tif]

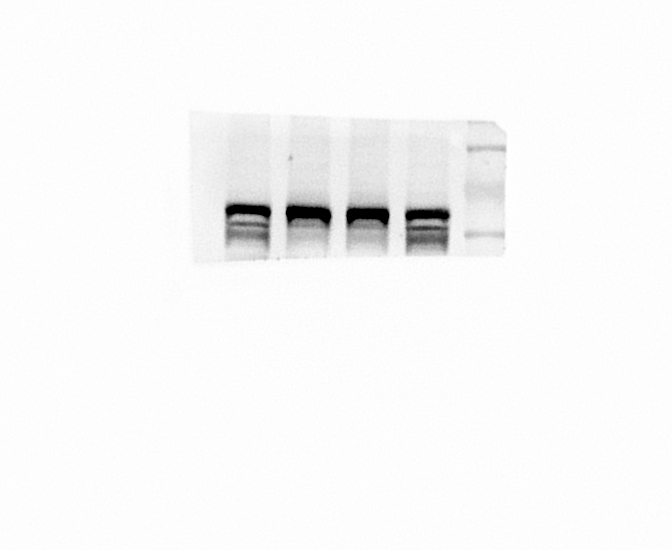

Supplement: Figure 8—source data 1. [file elife-94765-fig8-data1.zip › Figure 8-source data 1/Figure 8l/SRC/SRC_4.tif]

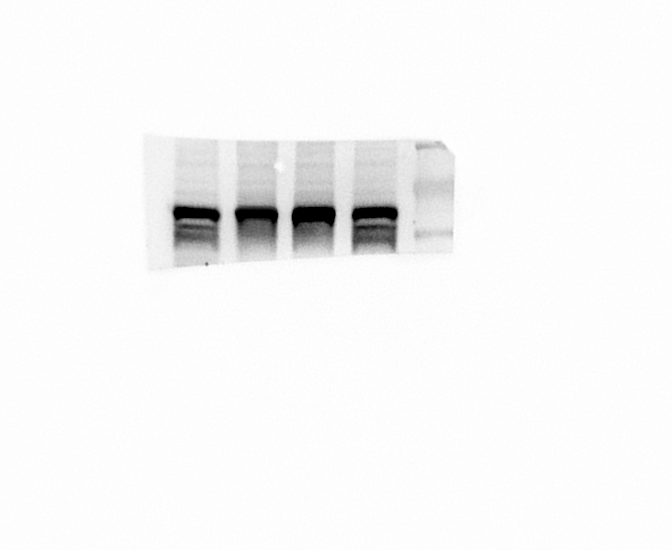

Supplement: Figure 8—source data 1. [file elife-94765-fig8-data1.zip › Figure 8-source data 1/Figure 8l/SRC/SRC_3.tif]

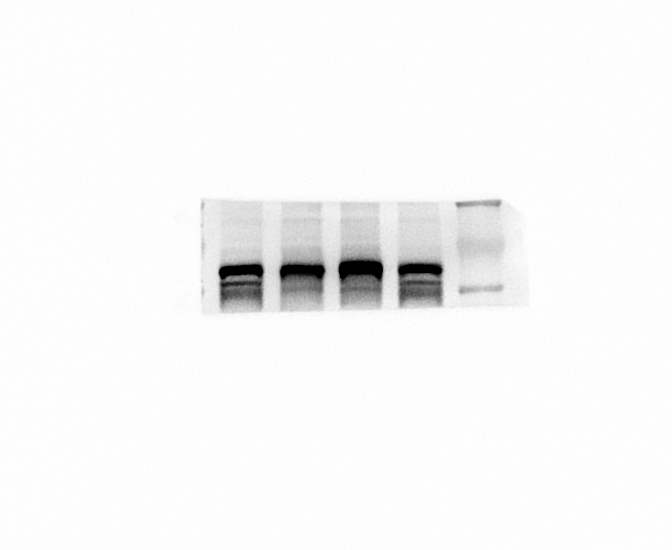

Supplement: Figure 8—source data 1. [file elife-94765-fig8-data1.zip › Figure 8-source data 1/Figure 8l/SRC/SRC-1.tif]

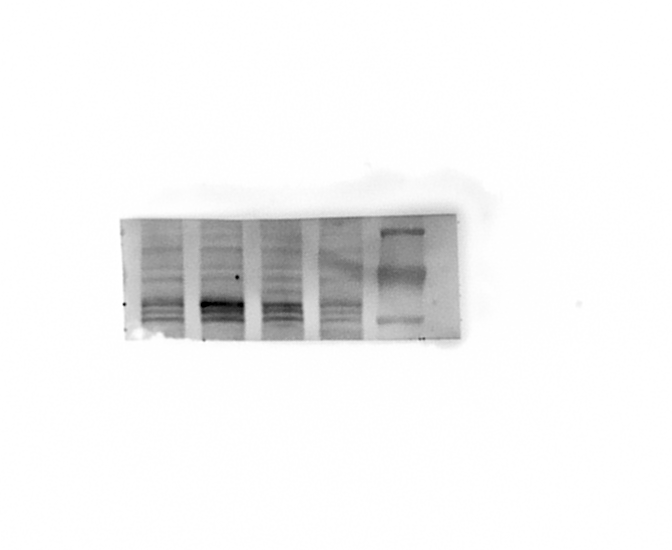

Supplement: Figure 8—source data 1. [file elife-94765-fig8-data1.zip › Figure 8-source data 1/Figure 8l/SRC/P_SRC_3.tif]

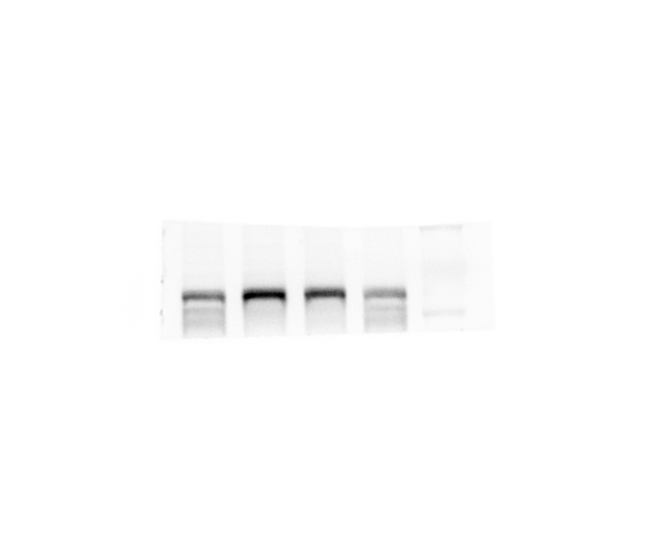

Supplement: Figure 8—source data 1. [file elife-94765-fig8-data1.zip › Figure 8-source data 1/Figure 8l/SRC/p-src-4.tif]

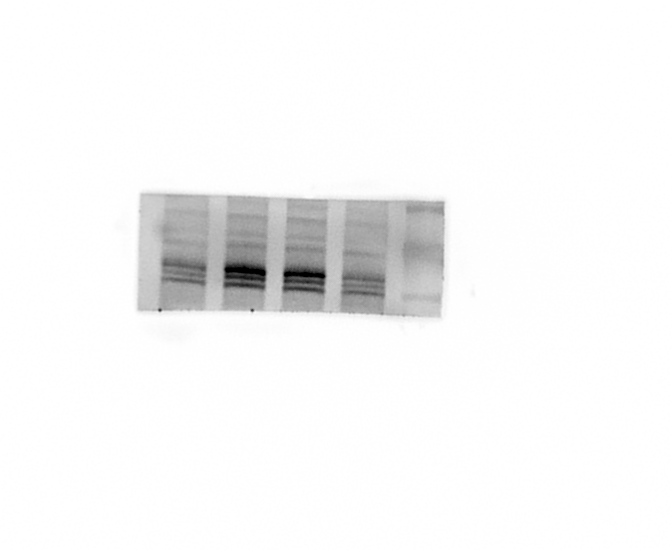

Supplement: Figure 8—source data 1. [file elife-94765-fig8-data1.zip › Figure 8-source data 1/Figure 8l/SRC/P_SRC-1.tif]

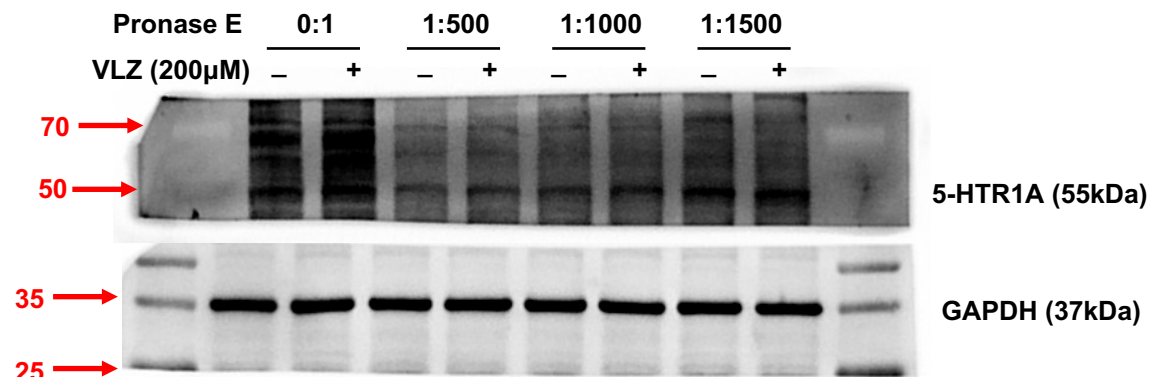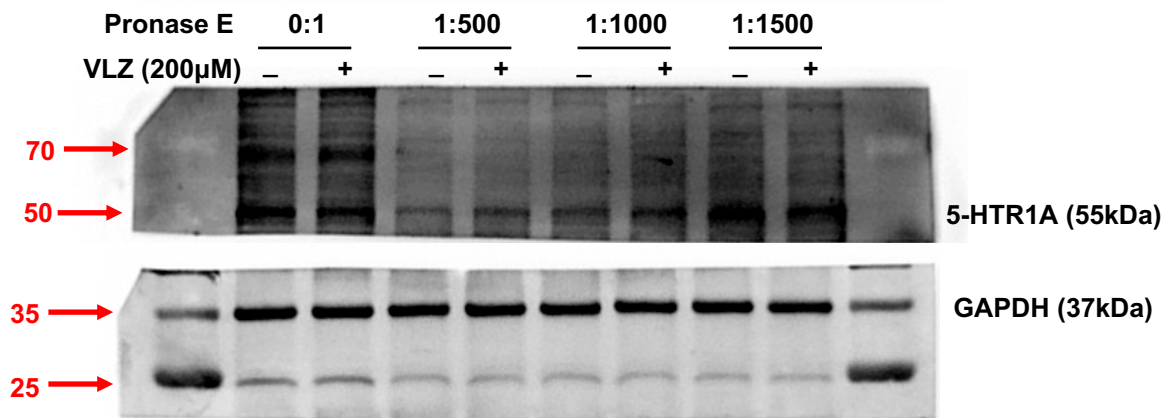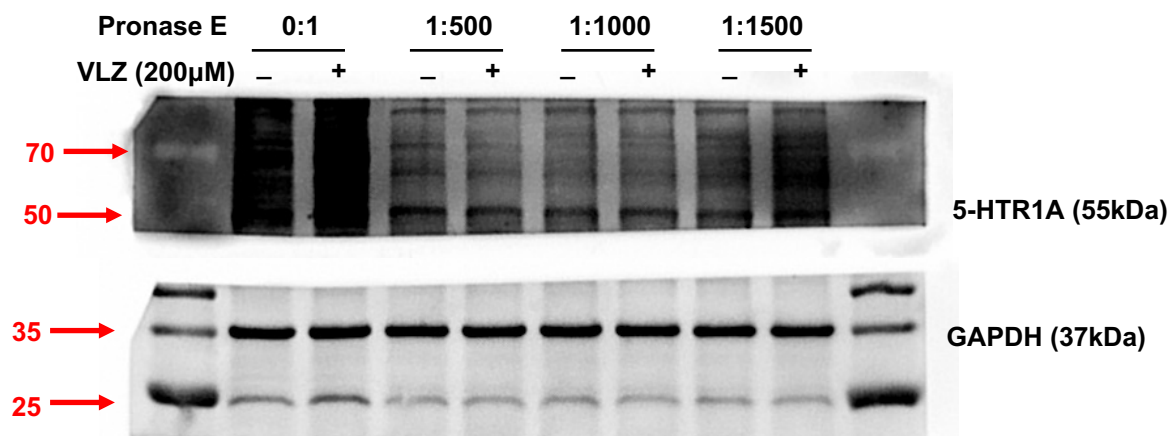

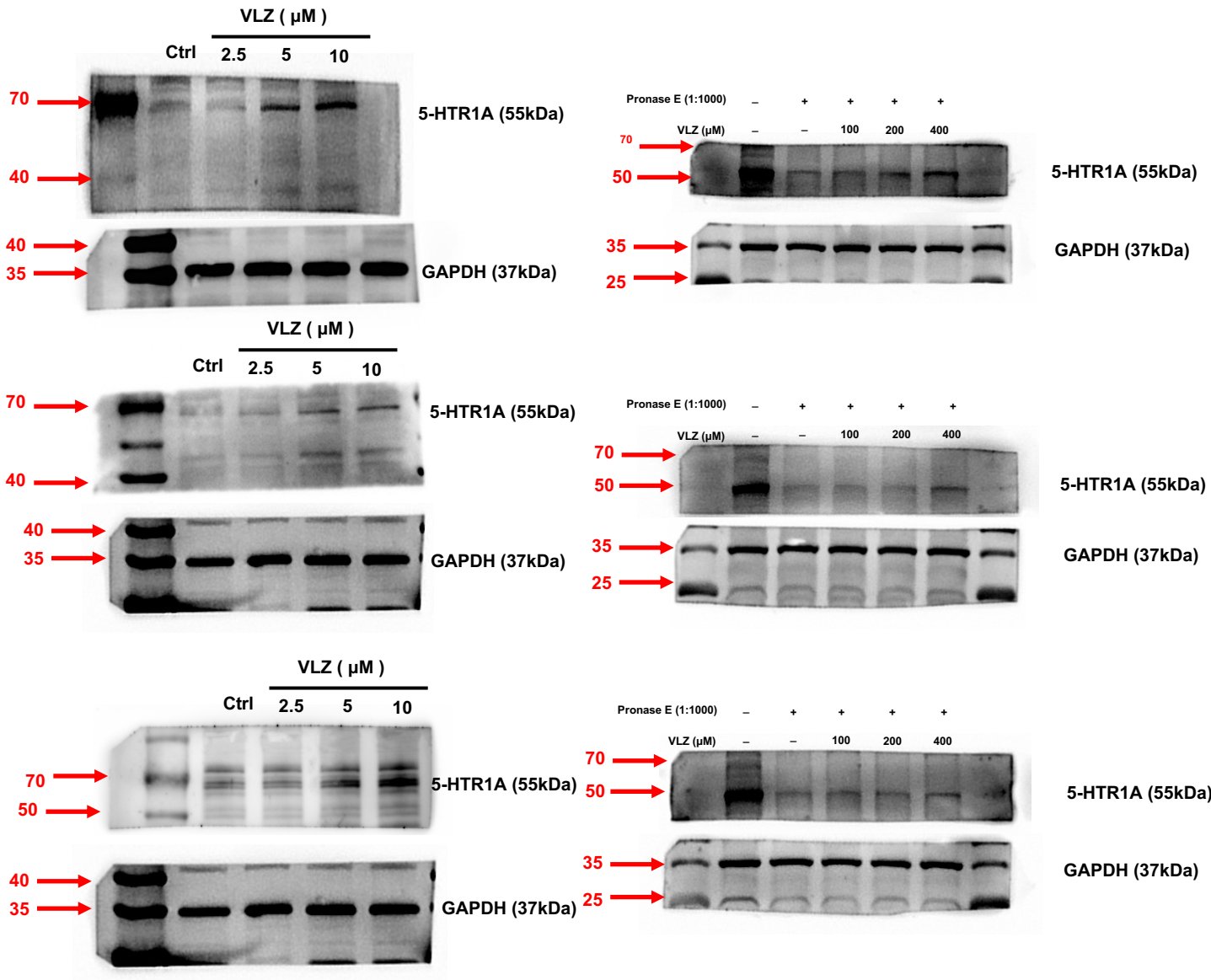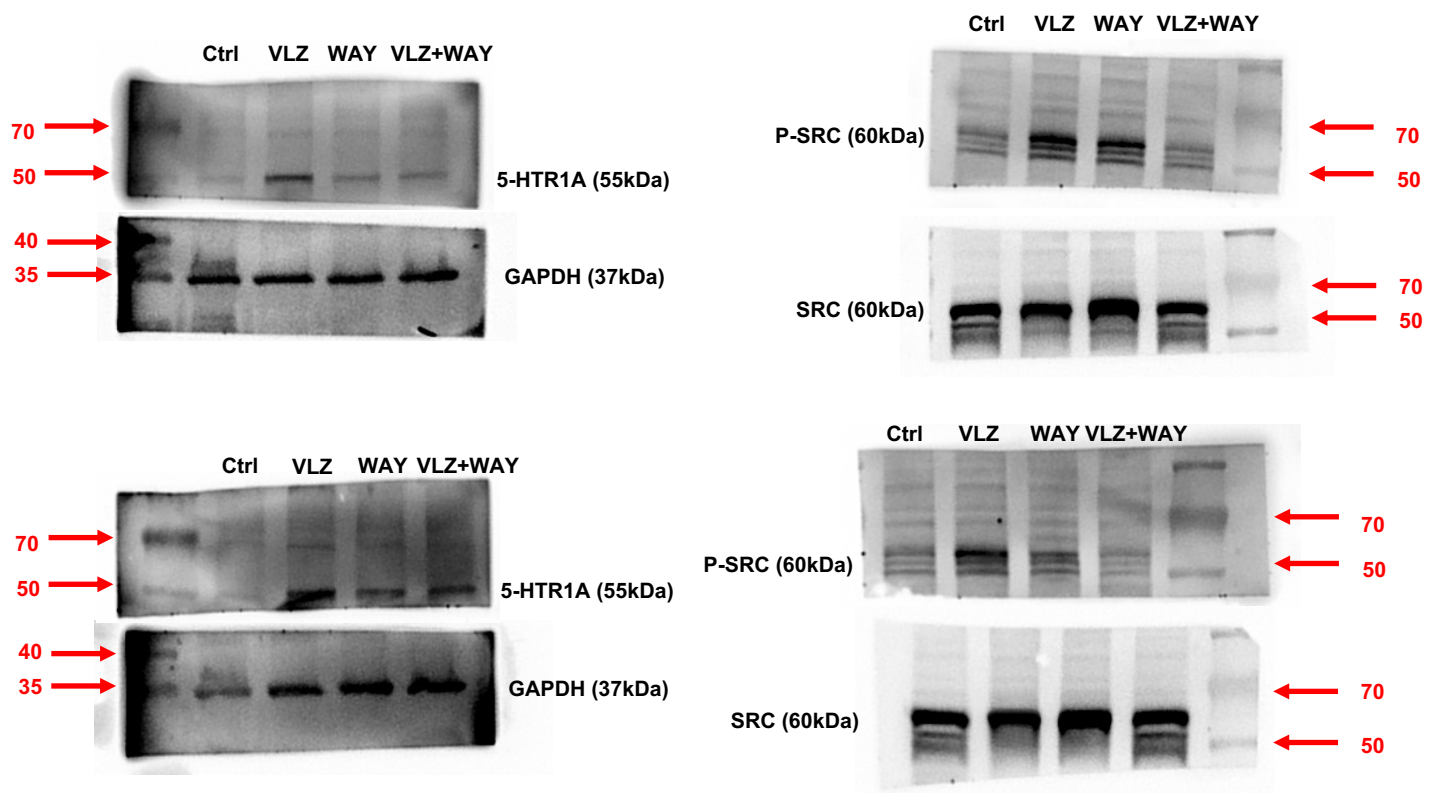

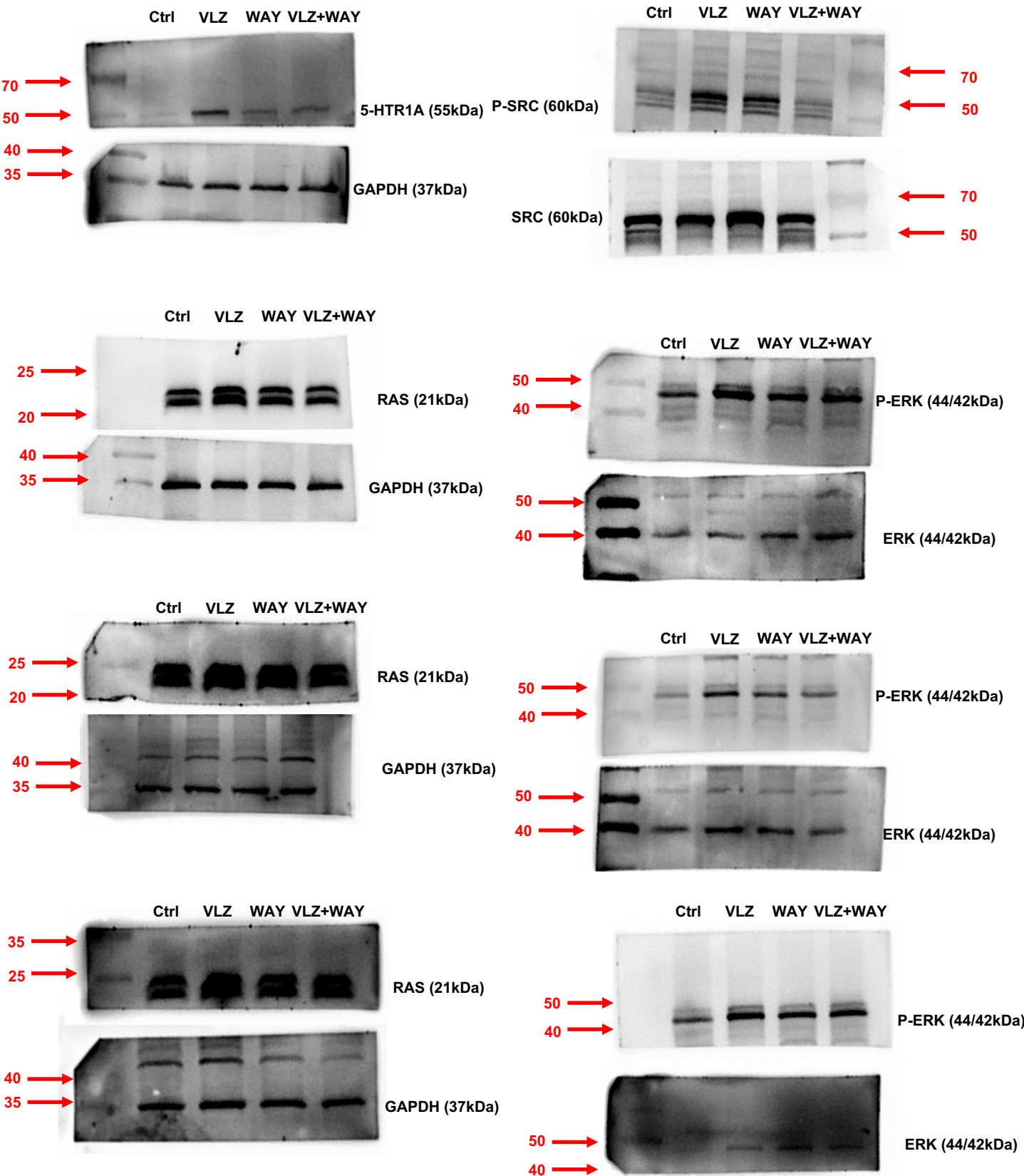

Supplement: Figure 8—source data 2. [file elife-94765-fig8-data2.pdf]
